# ZNF32 inhibits autophagy through the mTOR pathway and protects MCF-7 cells from stimulus-induced cell death

**DOI:** 10.1038/srep09288

**Published:** 2015-03-19

**Authors:** Yanyan Li, Le Zhang, Kai Li, Jun Li, Rong Xiang, Jie Zhang, Hongjiang Li, Yan Xu, Yuyan Wei, Junping Gao, Ping Lin, Yuquan Wei

**Affiliations:** 1 Division of Experimental Oncology, State Key Laboratory of Biotherapy/Collaborative Innovation Center for Biotherapy, West China Hospital, Sichuan University, Chengdu, China; 2 Department of clinical medicine, School of Medicine/Collaborative Innovation Center for Biotherapy, Nankai University, Tianjin, China; 3 Department of Thyroid and Breast Surgery, West China Hospital, Sichuan University, Chengdu, China; 4 Biorepository, State Key Laboratory of Biotherapy/Collaborative Innovation Center for Biotherapy, West China Hospital, Sichuan University, Chengdu, China; 5 Division of Cancer Biotherapy, State Key Laboratory of Biotherapy/Collaborative Innovation Center for Biotherapy, West China Hospital, Sichuan University, Chengdu, China

## Abstract

ZNF32 is a recently identified zinc finger protein and its functions remain largely unknown. Autophagy has been shown to affect cell proliferation and survival. Here, we innovatively show the effect of ZNF32 on cell autophagy and autophagy-associated cell death in breast carcinoma cells and also elucidate its underlying mechanisms. We examined the autophagic activity and LC3 II expression in human carcinoma cell lines with increased or decreased ZNF32 expression. Pharmacological inhibition (rapamycin) or activation (EGF) assays were used to investigate the function of the AKT/mTOR pathway during this process. H_2_O_2_- and diamide-induced MCF-7 cell death models were used to elucidate the role of ZNF32-associated autophagy in breast carcinoma cell death. Our results show that increasing ZNF32 expression in MCF-7 cells inhibits autophagy initiation by activating the AKT/mTOR pathway, and further reduced autophagy-associated cell death and maintained MCF-7 cell survival. Conversely, impairing ZNF32 expression by transfecting ZNF32 siRNA strongly promoted autophagy, further augmenting autophagy-associated cell death. Furthermore, correlations between ZNF32 and autophagy were observed in both MCF-7 xenograft tumors and in breast cancer patients. In conclusion, ZNF32 acts as an effective autophagy inhibitor to protect breast cancer cells from excessive stimulus-autophagy-induced cell death.

Human Zinc Finger Protein 32 (ZNF32), a recently discovered zinc finger protein, maps to chromosome 10q^23–24^. ZNF32 is reported to be a transcription factor belonging to the Kruppel-related zinc finger family^1^. Based on our previous study, mouse Zinc Finger Protein 637 (ZFP637), the homologous gene of human ZNF32, was implicated in promoting EMT-6 (a mouse breast carcinoma cell line) proliferation^2^ and preventing C2C12 (a mouse myoblast cell line) differentiation^3^. However, the exact functions of human ZNF32 remain largely unknown. Potential target genes of ZNF32 remain under investigation. The mechanisms of ZNF32-associated transcription regulation and its downstream events also remain unclear.

Autophagy is generally considered to be a process of cellular self-renewal, including the formation of autophagosomes and the degradation of organelles and cytosolic macromolecules^4^. With the assistance of autophagy-related gene 5–12 (Atg5-Atg12) conjugates, the precursor of microtubule-associated protein 1 light chain 3 alpha (LC3), a homolog protein of the yeast autophagy marker Atg8 and an interactive protein of the microtubule-associated protein 1 (MAP-1) family^5^,^6^,^7^, is cleaved to form cytosolic LC3 I, which further conjugates with phosphatidylethanolamine to produce the isolation membrane-associated LC3 II^8^,^9^. Dysfunctional organelles or misfolded proteins are escorted into autophagosomes for lysosomal degradation after binding to a substrate receptor^10^,^11^. However, excessive autophagic activity has been shown to result in cell death, which has been designated as type II programmed cell death or autophagy-associated cell death. In other words, basal autophagy has been shown to barely affect cell death, but the excessive autophagy induced by intense stimuli usually results in cell damage or even cell death. In recent years, increasing evidence has suggested that the autophagy of cancer cells is involved in cancer growth and progression^12^. Indeed, autophagy, a double-edged sword, has been reported to differentially influence cancer cell fate in different cell types and under different stimulus intensities^13^,^14^,^15^. On the one hand, autophagy can protect cancer cells from unfavorable growth condition and further attenuate the efficiency of anticancer drugs^16^. On the other hand, some reports have indicated that autophagy-associated cell death decreases cancer cell viability and enhances chemotherapy-associated anticancer activity^17^,^18^.

The formation of an autophagosome has been conventionally regarded as the initial step of autophagy. Currently, two major signaling pathways have been shown to be involved in this process^19^. In mammalian cells, autophagy initiation can be induced via the phosphoinositide 3-kinase/Protein Kinase B/mammalian target of rapamycin (PI3K/AKT/mTOR) pathway^20^, Moreover, nutrients have also been shown to regulate the initiation of autophagy through the serine/threonine kinase 11 (STK11, LKB1)/AMP-activated protein kinase (AMPK)/mTOR pathway^21^,^22^. Conversely, the B-cell CLL/lymphoma 2 (Bcl-2) family, which are anti-apoptotic proteins, exerts an opposite effect on autophagy initiation via two different pathways. First, Bcl-2 can prevent Beclin-1 from binding to PI3KCIII (class III PI3K) and then inhibit autophagy initiation through the PI3K/AKT/mTOR pathway^20^,^23^. Second, the Bcl-2 family can inhibit Beclin-1 function, which elevates p27 and Atg5 expression and facilitate autophagy through the LKB1/AMPK/mTOR pathway^21^,^22^.

Autophagy is closely related to cancer survival^24^. It remains unknown whether human ZNF32 modulates autophagic activity in carcinoma cells and affects cell viability. The underlying mechanisms of ZNF32-associated autophagy also remain unknown. Breast cancer causes high morbidity in women. Consequently, we aimed to investigate the effect of ZNF32 on breast cancer autophagy and viability and elucidate the underlying molecular mechanisms. Furthermore, we also analyzed the correlations between ZNF32 and autophagy in both xenograft tumors and in breast carcinoma patients.

## Results

### ZNF32 reduces autophagosome formation and inhibits autophagy initiation in MCF-7 cells

**Figure 1:**
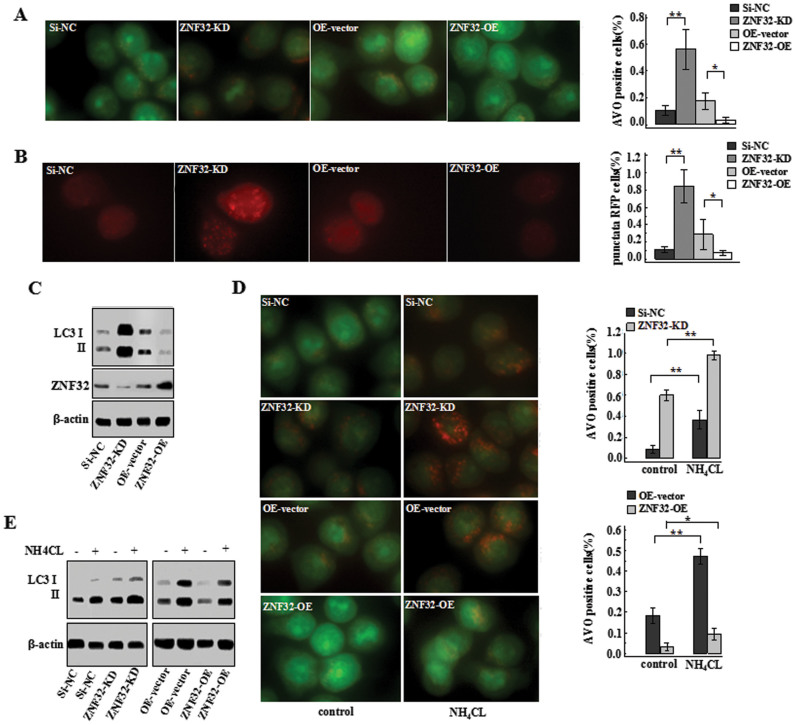
ZNF32 reduces autophagosome formation and inhibits autophagy initiation in MCF-7 cells with ZNF32 knock down (ZNF32-KD) or overexpression (ZNF32-OE). (A) Images of acridine orange (AO) staining and (B) RFP-LC3 transfection in cells as detected using fluorescence microscopy are shown. (C) LC3 II expression levels were measured using western blot analysis. (D) AO staining and (E) LC3 II expression in cells treated with 20 mmol/L NH_4_CL for 24 h. β-actin was used as a loading control. The full size blots were shown in the Supplementary Figure S4 and band of interest is indicated with an arrow.

To investigate the effect of ZNF32 on autophagy in breast cancer cells, ZNF32 siRNA and expression plasmids were introduced into a human breast carcinoma cell line (MCF-7). Acidic vesicular organelles (AVO) were visualized using AO staining (details described in Materials and methods). Strikingly, increasing ZNF32 expression in MCF-7 cells significantly reduced the percentage of AVO-positive cells. By contrast, interfering with ZNF32 expression increased the mean AVO-positive cell number (Fig. 1A). Additionally, we evaluated LC3 to further verify autophagosome formation. Plasmids expressing red fluorescent protein RFP-LC3-fusion protein were transfected into cells to label cytoplasmic autophagosomes^25^. Consistent with the previous result, a reduction in positive cells with punctiform aggregates was observed in the ZNF32-overexpressing cells, whereas more positive cells with punctiform aggregates were observed in ZNF32 knockdown cells (Fig. 1B). Moreover, a western blot analysis further verified that the expression of LC3 II, the lipidated form of LC3 associated with the autophagosomal membrane, decreased after ZNF32 overexpression. Conversely, LC3 II expression was higher in ZNF32-deficient cells (Fig. 1C). The same phenomenon was also observed in other breast cancer cell lines, such as SK-BR-3 and MDA-MB-231 (Fig. S1). In conclusion, these findings indicate that ZNF32 effectively decreases the autophagosome number and influences the autophagic process in breast carcinoma cells.

The quantity of autophagosomes is mainly determined by two independent processes of autophagy, which are autophagosome formation and autophagosome-lysosome fusion for subsequent degradation. Consequently, we examined which process was directly involved in ZNF32-associated regulation. To identify the precise process, NH_4_Cl was used to block autophagosome-lysosome fusion and autophagolysosome degradation^26^. Surprisingly, preventing the degradation of autophagosome adequately restored the number of AVO-positive cells (Fig. 1D) and the LC3 II level (Fig. 1E) in both the ZNF32-deficient and overexpressing cells. These results show that ZNF32 suppresses autophagy initiation whether autophagosome-lysosome fusion is allowed.

### ZNF32 inhibits autophagy initiation by activating the AKT/mTOR pathway

**Figure 2:**
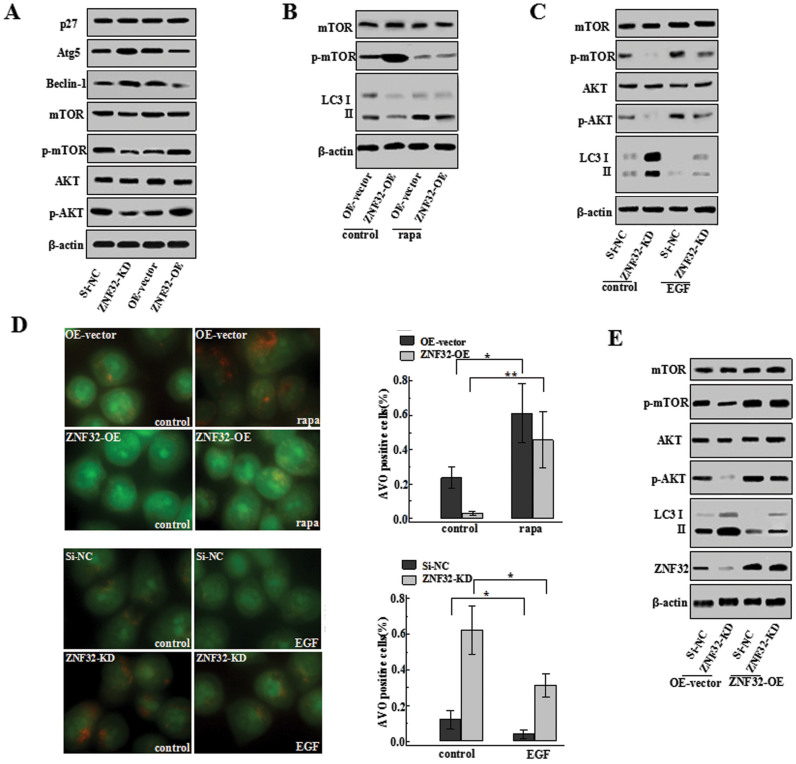
ZNF32 inhibits autophagy initiation by activating the AKT/mTOR pathway. (A) Effects of ZNF32-KD or ZNF32-OE on p27, Atg5, Beclin-1, p-AKT and p-mTOR expression. (B) mTOR activation in MCF-7 cells with ZNF32 overexpression after a 24 h treatment with 100 nmol/L rapa was determined using western blot analysis. (C) Phosphorylation of mTOR and AKT in MCF-7 cells with ZNF32 overexpression or knockdown after a 24 h treatment with 1 ng/ml EGF. (D) AO staining in MCF-7 ZNF32-KD or ZNF32-OE cells after 24 h treatment with 100 nmol/L rapa or 1 ng/ml EGF was detected using fluorescence microscopy. (E) ZNF32 expression rescues AKT/mTOR activation and LC3 II expression in ZNF32 knockdown cells. β-actin was used as a loading control. The full size blots were shown in the Supplementary Figure S5 and band of interest is indicated with an arrow.

ZNF32 has been shown to be involved in the initial step of autophagy. However, the underlying signaling pathway through which this generally accepted transcription factor regulates autophagosome formation required further investigation. Two classic signaling pathways have been implicated in mediating the initiation of autophagy: the Beclin-1-dependent p27/Atg5 and Beclin-1-dependent AKT/mTOR pathways. Beclin-1 has been reported to participate in both pathways. To investigate the mechanism in which ZNF32 affects autophagy, we first examined the expression of Beclin-1 in MCF-7 cells with modified ZNF32 expression. Similar to autophagy initiation, Beclin-1 expression was significantly suppressed in ZNF32-overexpressing cells and was increased in ZNF32-deficient cells (Fig. 2A). To further clarify which downstream pathway might be involved, we examined the expression of the key molecules in the both pathways. We found that although the expression levels of Beclin-1 and Atg5 were increased in the ZNF32-deficient MCF-7 cells, the expression level of p27 remained unchanged (Fig. 2A). This finding suggests that the p27-associated pathway is not involved in ZNF32-associated autophagy.

The Beclin-1-dependent AKT/mTOR pathway is another mediator of autophagy initiation. To determine whether this pathway is involved in ZNF32-regulated autophagy, the activation of AKT and mTOR was evaluated after increasing or decreasing ZNF32 expression in MCF-7 cells. Interestingly, increasing ZNF32 expression clearly promoted the phosphorylation of both AKT and mTOR (Fig. 2A). It is widely accepted that the AKT/mTOR pathway inhibits autophagy initiation. Therefore, it was hypothesized that an increase in ZNF32 can induce AKT/mTOR activation, which would further interfere with autophagy initiation. To test this hypothesis, rapamycin (rapa), a classic mTOR inhibitor^27^, was used to investigate the effect of mTOR on ZNF32-associated autophagy. Consistent with our hypothesis, inhibiting mTOR activation rescued ZNF32-induced autophagy deficiency (Fig. 2B, D). Furthermore, the EGF activation of the AKT/mTOR pathway reduced LC3 II production and inhibited autophagosome formation in ZNF32-deficient MCF-7 cells (Fig. 2C, D). Additionally, we also rescued ZNF32 expression in the ZNF32-knockdown MCF-7 cells to further verify its function in autophagy (Fig. 2E). These results demonstrate that ZNF32 can activate the AKT/mTOR pathway, suppressing autophagy initiation.

### ZNF32-associated autophagy participates in H_2_O_2_- and diamide-induced cell death

**Figure 3:**
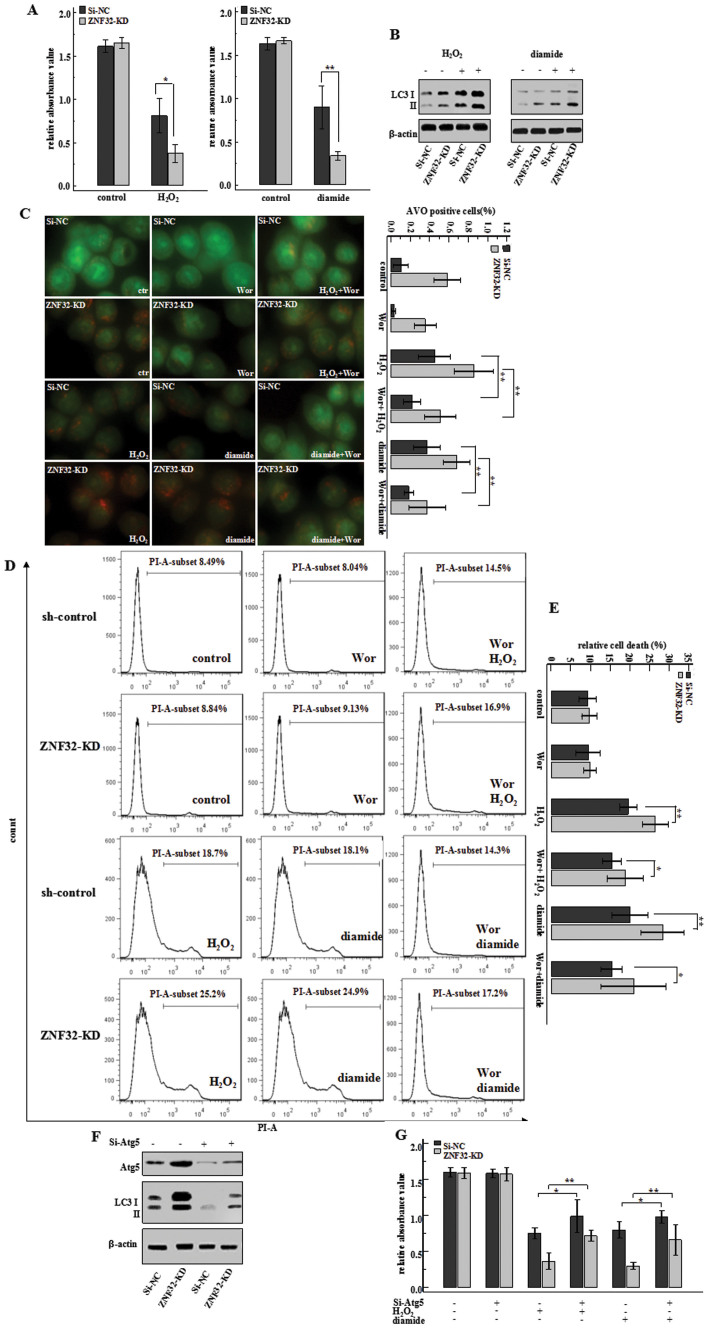
ZNF32-associated autophagy participates in H_2_O_2_-and diamide- induced cell death. (A) MTT assay showing the effect of ZNF32 on cell viability after 24 h treatment with H_2_O_2_ (700 μmol/L) or diamide (500 μmol/L). (B) LC3 II expression after a 24 h treatment with H_2_O_2_ or diamide in ZNF32-KD cells. (C) Images of AO staining in ZNF32-KD cells after a 24 h treatment with H_2_O_2_ or diamide with a 3 h pre-treatment with or without Wor (100 nmol/L). Wor has been shown to diminish spontaneous autophagy (the upper two of the 2^nd^ vertical row) and reduced stimulus-triggered autophagy (the 3^rd^ vertical row). (D) Flow cytometry assay showing the effect of ZNF32-KD on cell death after a 24 h treatment with H_2_O_2_ or diamide with a 3 h pre-treatment with or without Wor (100 nmol/L). (E) The bar results are shown. (F) Atg5 interference efficiency and LC3II expression were detected using western blot analysis. β-actin was used as a loading control. The full size blots were shown in the Supplementary Figure S6 and band of interest is indicated with an arrow. (G) MTT assay showing the effect of si-Atg5 on MCF-7 cell viability after a 24 h treatment with H_2_O_2_ or diamide.

Although closely associated with autophagy, ZNF32 has demonstrated no effects on cell viability without stimulation (Fig. 3A, D control group). Autophagy has been reported to mediate stimulus-induced cell death^28^. First, to verify the effect of autophagy on H_2_O_2_- and diamide-induced cell death, high doses of H_2_O_2_ (700 umol/L) and diamide (500 umol/L) were used. A MTT assay was used to evaluate cell death (Fig. 3A). The results showed that a ZNF32 deficiency augmented this stimulus-induced cell death. Indeed, with H_2_O_2_ or diamide stimulation, the LC3 II expression level increased in both si-NC and ZNF32 knockdown cells, but the increases appeared to be more dramatic in the ZNF32 knockdown cells (Fig. 3B). Consequently, we proposed that ZNF32-regulated autophagic activity could further affect H_2_O_2_- and diamide-induced cell death. To test this proposal, we first examined the autophagic activity in ZNF32-modified MCF-7 cells treated with H_2_O_2_ and diamide. Consistent with our previous observation, ZNF32-knockdown MCF-7 cells demonstrated the highest level of autophagic activity after stimulation with H_2_O_2_ and diamide (Fig. 3C). Moreover, Wor, which is a specific inhibitor of autophagy that has been shown to diminish spontaneous autophagy (Fig. 3C the upper two of the 2^nd^ vertical row), also reduced stimulus-triggered autophagy (Fig. 3C the 3^rd^ vertical row); the bar results are shown on the right. Interestingly, as shown in Fig. 3D and E, Wor partially reduced the H_2_O_2_- and diamide-induced cell death in these cells and reduced the difference between the ZNF32-knockdown and corresponding control cells (Fig. 3D the 3^rd^ vertical row). Atg5 is thought to be an essential gene for autophagy^29^,^30^ and has been shown to be involved in the formation of autophagosome^31^. Because of the controversial role of Wor and considering its unrelated side effects, Atg5 siRNA was used. Silencing Atg5 clearly decreased LC3 II expression (Fig. 3F) and also reduced the difference in H_2_O_2_- and diamide-induced viability between the ZNF32-knockdown and corresponding control cells (Fig. 3G). Overall, these results indicate that ZNF32-associated autophagy facilitates stimulus-induced cell death to some extent.

### AKT/mTOR pathway is involved in the ZNF32-autophagy-cell death axis

**Figure 4:**
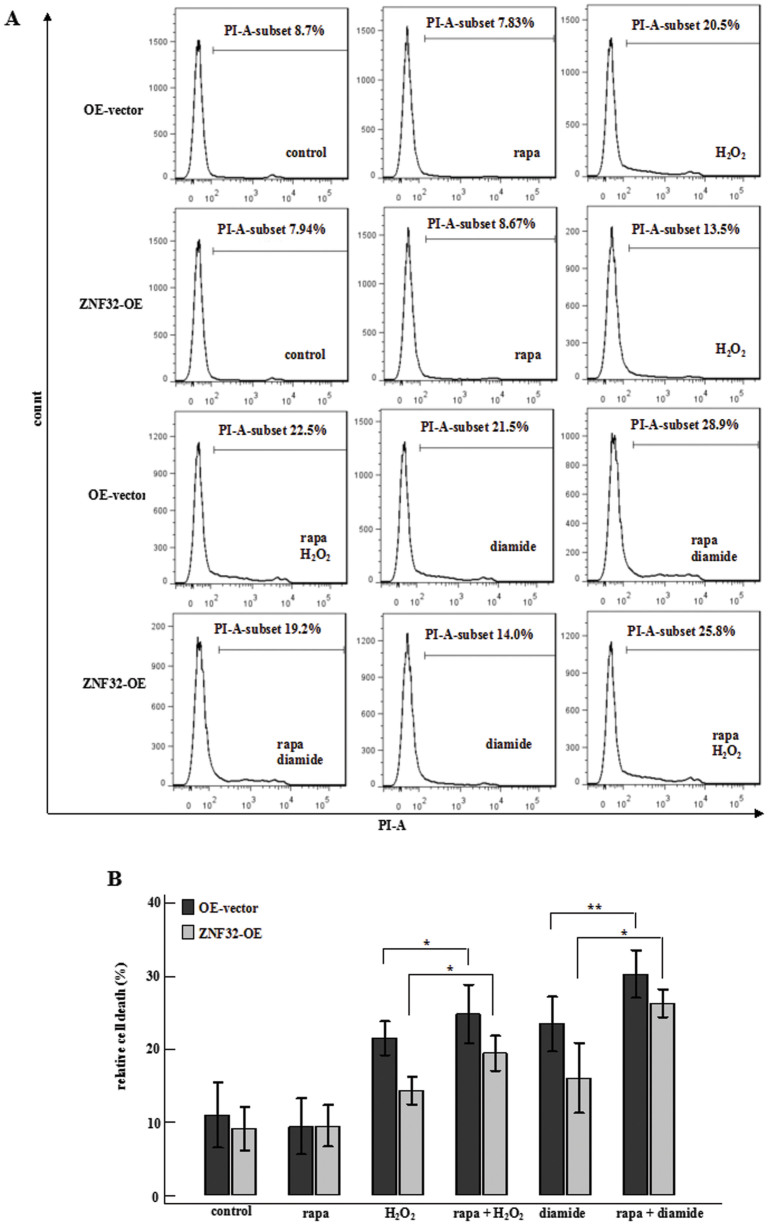
AKT/mTOR pathway is involved in the ZNF32-autophagy-cell death axis. (A) Flow cytometry reveals the effect of ZNF32 knockdown on cell death after a 24 h treatment with H_2_O_2_ (700 μmol/L) or diamide (500 μmol/L). rapa (100 nmol/L) was used to inhibit mTOR activation in a 3 h pre-treatment before added H_2_O_2_ or diamide. (B) The bar results are shown.

The AKT/mTOR pathway has been shown to be involved in ZNF32-associated autophagy, which may affect stimulus-induced cell death. Therefore, we intended to further verify that the AKT/mTOR pathway participates in the ZNF32-autophagy-cell death axis. Strikingly, in ZNF32-overexpressig MCF-7 cells, inhibiting mTOR activation with rapa partially rescued the autophagic activity (Fig. 2B and Fig. 2D) and slightly increased H_2_O_2_- and diamide-induced cell death (Fig. 4A and B). Moreover, EGF was also used to activate the AKT/mTOR signaling pathway in ZNF32-deficient MCF-7 cells. Similarly, activation of the AKT/mTOR pathway inhibited autophagy and partially counteracted the ZNF32-enhanced cell death after H_2_O_2_ and diamide treatment (Fig. S2). These findings suggest that ZNF32 inhibits stimulus-induced autophagy and cell death at least partially through AKT/mTOR activation.

### ZNF32 is correlated with autophagy in both xenograft tumor-loaded mice and in breast cancer patients

**Figure 5:**
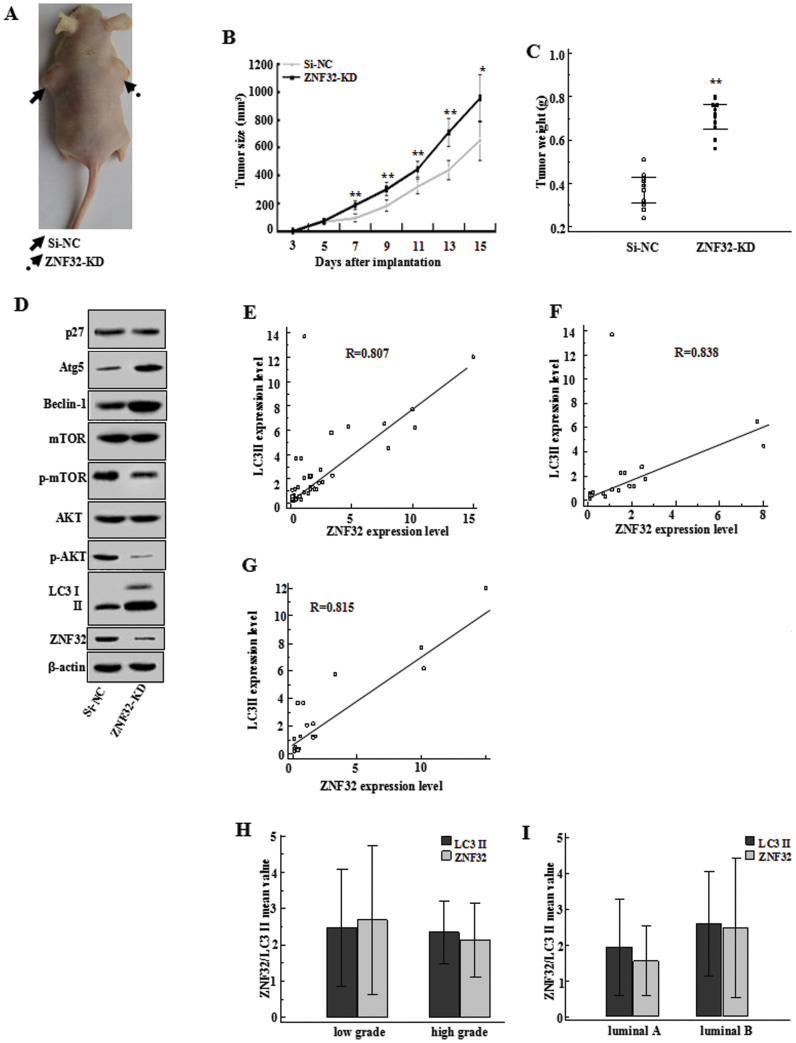
ZNF32 is correlated with autophagy in both xenograft tumor-loaded mice and in breast cancer patients. (A) A total of 5 × 10^6^ viable MCF-7 si-NC or ZNF-32 KD cells were implanted subcutaneously into nude mice. Pictures of the treatment group are shown. Mice were killed 2 weeks after implantation. (B) Tumor growth (volume) from the 3^rd^ to 15^th^ day after implantation. (C) Tumor weight (g) was measured on the 15th day after implantation. (D) Western blot results showing the expression levels of some autophagy related markers in tumor tissues. β-actin was used as a loading control. The full size blots were shown in the Supplementary Figure S6 and band of interest is indicated with an arrow. Correlation analysis between ZNF32 and LC3 II in (E) the total (F) luminal A subtype and (G) luminal B subtype breast cancer patient samples. Expressions of ZNF32 and LC-3 II in (H) low or high pathological grade (I) luminal A or luminal B subtype of breast cancer samples.

To investigate the impact of ZNF32 on autophagy in vivo, a MCF-7-bearing mouse model was constructed. Interestingly, although unable to directly affect cell viability in vitro, ZNF32 was observed to regulate tumor growth in vivo (Fig. 5A). Surprisingly, inconsistent with our prediction, we found that ZNF32 knockdown of MCF-7 cells could form tumor masses that were larger in volume and weight in the null mouse models (Fig. 5B, C). Next, we examined the autophagic status of the xenograft tumors. Consistent with the previous in vitro results, decreasing ZNF32 expression also promoted autophagy in the xenograft tumors. As shown in Figure 5D, LC3 II expression was higher in the ZNF32-deficient tumors. Additionally, AKT/mTOR activation was inhibited after interfering with ZNF32 expression. Moreover, the p27 pathway appeared to remain unchanged (Fig. 5D). These results imply that ZNF32 also modulates autophagic activity via the AKT/mTOR pathway in vivo. However, its impact on tumor growth is complicated.

To better understand the relationship between ZNF32 and autophagy, we collected breast cancer samples from 49 cases and used western blot analysis to detect the ZNF32 and LC3 II expression levels. Based on the ZNF32 and LC3 II expression levels, we conducted a correlation analysis (Fig. 5E). Surprisingly, we found that ZNF32 expression was positively correlated with LC3 II expression in breast cancer patients (R = 0.807). Considering that MCF-7 is a luminal A subtype (ER and PR positive, Her-2 negative) cell line and that the luminal subtype is common,we divided these samples into 23 cases of luminal A subtype, 20 cases of luminal B subtype (ER and PR positive, Her-2 positive) and 4 Her-2-enrich cases, 1 basal-like case, 2 normal-like cases, based on the intrinsic molecular subtypes of breast cancer^32^,^33^,^34^. Consistent with the trends observed in the 49 cases overall, we found that ZNF32 expression was positively correlated with LC3 II expression in both subtypes of breast cancer samples (Fig. 5F luminal A subtype and Fig. 5G luminal B phenotype). The sample sizes of the other group cases were too small to conduct a correlation analysis. Somehow, this result seems to contradict the observations made in vitro and in the mouse models. These findings suggest that more complicated factors may also be involved in the ZNF32-associated regulation of autophagy in cancer patients. Furthermore, we compared the ZNF32 and LC3 II expression levels in two groups of patients with high and low pathological grades of breast cancer (see details in Materials and methods, Elston-Ellis modification of the Scarff-Bloom-Richardson Grading System). However, there were no significant differences in the ZNF32 and LC3 II expression levels between these two pathological grade groups (Fig. 5H), even in the luminal A and luminal B subtype breast cancer samples (Fig. S3). We also compared the ZNF32 and LC3 II expression levels in the luminal A and luminal B subtype breast cancer samples (Fig. 5I). There were no significant differences in the ZNF32 and LC3 II expression levels between the two groups, suggesting that the trend in ZNF32 and LC3 II expression may be irrelevant to the gene expression-based “intrinsic” subtype. Although there was no significant difference between the two pathological grade groups, possibly because of the small sample size or other unknown factors, we observed an accelerated growth of ZNF32-deficient breast cancer cells in the mouse models. The relationship between ZNF32 and the pathological grade of breast cancer may be quite complicated inside organisms. However, it was shown that the ZNF32 and LC3 II expression levels are correlated in breast carcinoma patients.

## Discussion

Currently, resistance to general therapies, including chemotherapy, radiotherapy and immunotherapy, perplexes oncologists and clinicians worldwide. How to overcome this obstacle is a wide concern. Clarifying the underlying mechanisms may greatly improve the efficiency of cancer therapies. Recently, autophagy has been shown to contribute to the therapy-associated resistance of cancer^35^. However, the exact role of cancer cell autophagy in resistance to treatment is still controversial. ZNF32, a recently discovered transcription factor, has been shown to be closely associated with oxidative stress. In this study, we elucidated a novel function of ZNF32 in autophagy and autophagy-associated cell death. ZNF32 was shown to activate the AKT/mTOR pathway, which subsequently decreases the severe autophagic activity in breast carcinoma cells. ZNF32 may protect breast cancer cells from excessive autophagy-associated cell death. Moreover, ZNF32 was also shown to be correlated with autophagy in xenograft tumor-loaded mice and cancer patients. Altogether, we indicate that ZNF32 may be a potential target for breast cancer treatment.

We found that ZNF32 inhibited the initiation of autophagy by activating the AKT/mTOR pathway. However, as a transcription factor and not a phosphokinase, ZNF32 cannot directly phosphorylate AKT/mTOR. There must be an unknown intrinsic link between ZNF32 and AKT/mTOR activation. The direct target genes of ZNF32 involved in AKT/mTOR activation still remain unknown. According to bioinformatics data, there may be a potential ZNF32 binding sequence in the PI3K promoter region. Perhaps, ZNF32 directly induces PI3K expression and activates AKT/mTOR. A further investigation is required to answer this question.

Based on our observations, even though interfering with ZNF32 expression augmented spontaneous autophagy in carcinoma cells, the viability of these cells remained unaffected. This finding suggests that the level of ZNF32-induced autophagy was lower than the autophagic threshold that induces cell death. Reactive oxygen species (ROS) have been widely recognized to mediate cancer development and progression. Indeed, H_2_O_2_ and diamide, the conventional ROS inducers, adequately promoted autophagy, decreasing the viability of cancer cells. In this case, ZNF32 prevented the excessive autophagic activity induced by ROS, reducing cell death. In ZNF32-defective carcinoma cells, which lack this protective mechanism, ROS may induce a higher level of autophagy and increase cancer cell death. It has been reported that the zinc-finger protein MCPIP-induced ROS causes an ER stress response, inducing autophagy^36^. Our data (unpublished) indicated that ZNF32 may affect the function of mitochondria. The change in mitochondria function may be one of the factors affecting autophagy. Perhaps ZNF32 also functions in organelles, such as the mitochondria or ER, to regulate autophagy. Whether ZNF32-associated autophagy affects cell death induced by factors other than ROS warrants additional investigation.

Although ZNF32 appears to reduce autophagy and increase cell viability in vitro, decreasing ZNF32 expression in xenograft tumors increased the mean volume and weight of the tumors in vivo. More interestingly, consistent with the in vitro results, the autophagic activity in the ZNF32-knockdown xenograft tumors appeared to be elevated according to the LC3 II expression pattern. Most unpredictably, we observed a positive correlation between ZNF32 and autophagy in breast cancer patients, which was inconsistent with our observations in vitro and in the mouse models. An analysis of the pathological grades of the breast cancer patient revealed that there was no difference in the ZNF32 and LC3 II expression levels, even between the luminal A and luminal B subtypes. Indeed, multiple factors, besides autophagy, may contribute to tumor growth in vivo. Some studies have reported that autophagy can promote cell death and make tumor cells more sensitive to stimulation^37^. Some studies, however, have reported different points of view^13^,^38^. Although we cannot attribute all the differences of tumor growth to autophagy, it may affect tumor growth to some extent as autophagy appears to be involved in tumorigenesis, cancer development and treatment outcome^39^,^40^. Because of the advances in cancer research, autophagy may act as a cancer suppressor or promoter depending on the different cell types, grades and therapeutic strategies^40^,^41^. Studies have reported that autophagy can promote or prevent apoptosis in the same cell type; the response is governed by the nature of the death stimulus and the compensatory changes in other forms of autophagy^28^,^42^. ZNF32 may regulate autophagy under more control in vivo. The diversity of the tumor microenvironment complicates the analysis of the in vivo mechanisms underlying ZNF32 regulation of tumor growth. Perhaps this diversity is why our in vitro, in vivo and patient sample results were not consistent. Explaining this inconsistency requires further investigation. We are further studying the complexity of ZNF32 regulation of tumor growth in vivo. All of the results indicated that ZNF32 is associated with breast cancer autophagy. We believe that ZNF32-associated gene therapy may improve the therapeutic efficiency of chemotherapy and benefit cancer patients. Until recently, several lines of evidence have indicated that ZNF32 may be involved in the diverse processes of cancer progression. Consequently, we faithfully recognize that a further investigation of ZNF32 functions in cancer biology would stimulate great advances in cancer diagnostics and therapeutics.

## Methods

### Cells, reagents and antibodies

The MCF-7, SK-BR-3 and MDA-MB-231 cell lines were maintained in complete Dulbecco's modified Eagle's medium (DMEM) and RPMI 1640 medium containing 10% FBS in humidified atmosphere at 37°C, 5% CO_2_. Wortmannin (Wor), NH_4_Cl, H_2_O_2_, rapamycin (rapa), diamide, epidermal growth factor (EGF) were purchased from Sigma-Aldrich (St Louis, MO, USA). The Cell Apoptosis Assays Kit was purchased from KaiJi (NanJing, China).

### Western blot analysis

Protein from cells or tissue samples was extracted using General Protein Extraction Reagent (Bioteke, Beijing, China) supplemented with 1% protease inhibitor. A total of 25 μg protein was loaded and separated on 12% SDS-PAGE and then transferred by electrophoresis to 0.45 μm polyvinyl difluoride membranes. The following antibodies were used: LC3, Atg5, p27, mTOR, phosphorylated mTOR (p-mTOR), AKT, and phosphorylated AKT (p-AKT), which were purchased from Cell Signaling Technology (CST, Danvers, MA, USA). β-actin antibody was purchased from ZhongShanJinQiao (BeiJing, China). All of the above-mentioned antibodies were used at a dilution ratio of 1:1000. ZNF32 antibody was produced and purified as previously described^43^ and used at a dilution of 1:50. The membrane was developed using Immobilon™ Western Chemiluminescent HRP Substrate (Millipore). Protein levels were quantified using scanning blots on a Gel Doc EZ imager (Bio-Rad, California, USA) and analyzing with Quantity One 1D image analysis software 4.4.0 (Bio-Rad, California, USA).

### Cell viability assay

Cells were plated onto 96-well plates and treated with H_2_O_2_ (700 umol/L) or diamide (500 umol/L) or the corresponding solvent control. Cell viability was measured using a MTT assay. The optical density was determined at 570 nm with a reference filter of 630 nm using an ELISA plate reader (Model 550; Bio-Rad). At least three independent experiments were conducted.

### Analysis of cell death

Cell death was evaluated using a propidium iodide (PI) staining Flow Cytometry analysis Kit (Kaiji, Nanjing, China) according to the manufacturer's instruction. The analysis was performed using a BD FACSAria (Franklin Lakes, NJ, USA). Individual fluorescent populations were determined using acquisition and analysis software (FlowJo 7.6). Triplicate independent experiments were conducted.

### Transfection

The pSG5-ZNF32 over-expression plasmid was constructed by inserting expanded ZNF32 cDNA (NCBI Reference Sequence: NM_006973.2) fragments into pSG5 vectors stored in our lab. RFP-LC3 was stored in our lab. ZNF32, Atg5 and non-target siRNA were purchased from Genepharm (Shanghai, China), ZNF32 siRNA: GUCAGAAAGGAAGCUUAAUTT (sense 5′-3′), Atg5 siRNA: AUCCCAUCCAGAGUUGCUUGUGAUC (sense 5′-3′), non-target siRNA: UUCUUCGAACGUGUCACGUTT (sense 5′-3′). Transfections were mediated by TurboFect Transfection Reagent (Thermo Fisher Scientific, Waltham, MA). Cells were pre-cultured in serum-free DMEM for 2 h before transfection. Then, 6 μg plasmid or 10 nmol siRNA was introduced into the cell using 6 μl transfection reagent according to the manufacturer's instructions. The punctiform aggregates and cells were counted at a final magnification of 400×. The percentage of positive cells with at least 5 punctiform aggregates was calculated; at least 600 cells were observed.

### Acridine orange (AO) staining

Acridine orange with membrane permeability spontaneously aggregated in acidic autophagosomes and exhibited punctate staining. It is conventionally used to visualize cytoplasmic autophagosome and detect autophagosome formation. To examine the formation of autophagosomes, cells were plated onto 48-well plates at a density of 3 × 10^4^ cells per well. Approximately 24 h later, the cells were rinsed with PBS three times. Then, the cells were treated with 1 μg/ml AO at 37°C for 30 min and rinsed with PBS three times. The formation of autophagosomes was observed and images were captured using an inverted fluorescent microscope (Nikon ECLIPSE TE 2000-U). The AO staining punctiform aggregates and the cells were counted at a final magnification of 400×. The percentage of AVO positive cells with at least 10 AVO aggregations was calculated; at least 600 cells were observed. Subsequently, a statistical analysis was performed. Triplicate independent experiments were conducted.

### Animals

BALB/c male nude mice were used at aged 6–8 weeks. A total of 5 × 10^6^ viable MCF-7 cells with low (ZNF32-KD) or normal (si-NC) ZNF32 expression were subcutaneously injected into the lateral backsides of the mouse. The tumor volume was recorded every two days from the 3rd to 15th day after implantation. All mice were killed 2 weeks later, and the xenograft tumors were collected and measured tumor weight (g). All experimental protocols were approved by the Committee on the Use of Live Animals in Teaching and Research of SiChuan University. The methods were conducted in accordance with the approved guidelines.

### Tissue samples from BCC patients

A total of 49 cases of human BCC samples were obtained from the Tumor Tissue Bank of West China Hospital. The diagnosis, pathological grade (Elston-Ellis modification of the Scarff-Bloom-Richardson Grading System) and the subtypes classification (luminal A, luminal B, Her-2-enrich cases, basal-like and normal-like) of the breast cancer for all samples were confirmed by a pathologist. To simplify the statistical analysis, we designated grades 1–2 as low grade and grade 3 as high grade. The tissues were snap-frozen in liquid nitrogen immediately after dissection and stored in liquid nitrogen until further assessment using western blot analysis. We calculated the gray value of ZNF32, LC3 II and β-actin, and then normalized the results by comparing ZNF32 or LC3 II with β-actin.

The study was approved by the local Ethical Committee on Human Experimentation of West China Hospital, Chengdu, and informed written consent was obtained from all the patients. Then, all of the collected samples were eligible for experimental purposes. The methods were conducted in accordance with the approved guidelines.

### Statistical analysis

Statistical analyses were performed with SPSS 13.0 (SPSS, Chicago, IL, USA). One-way analysis of variance test was used to evaluate the results of the MTT assay and AO staining. Correlation analysis was conducted to examine the relationship between ZNF32 expression and LC3 II expression in breast cancer patients. Student's t test was used to analyze the differences in the ZNF32 or LC3 II expression in breast cancer samples. The equality of variances was assured by Levene's test. *P* values < 0.05 were considered statistically significant. *Indicates that the *P* values were < 0.05 and >0.01, whereas **indicates *P* values < 0.01.

## Author Contributions

Y.L. carried out the research and drafted the manuscript. L.Z. revised the manuscript. K.L. and J.L. helped to feed and prepare the mice. R.X. and Y.W. kindly offered valuable suggestions about the experiment design. J.Z. provided some cell culture. H.L. and Y.X. provided human BCC samples. Y.W. and J.G. provided pSG5-ZNF32 over-expression plasmid and transfection. P.L. designed the experiments, analyzed the results and revised the manuscript. All authors reviewed the manuscript.

## Supplementary Material

Supplementary Information Supplementary Information
