# Supplementary material for: ZNF32 inhibits autophagy through the mTOR pathway and protects MCF-7 cells from stimulus-induced cell death
Source: Sci Rep. 2015 Mar 19;5:9288. doi: 10.1038/srep09288 (PMC4365391; doi:10.1038/srep09288)
Supplement: Supplementary Information [file srep09288-s1.doc]

**Supplementary Information**

**ZNF32 inhibits autophagy through the mTOR pathway and protects MCF-7 cells from stimulus-induced cell death**

Yanyan Li, Le Zhang, Kai Li, Jun Li, Rong Xiang, Jie Zhang, Hongjiang Li, Yan Xu, Yuyan Wei, Junping Gao, Ping Lin*and YuquanWei

- Supplementary Figure S1

- Supplementary Figure S2

- Supplementary Figure S3

- Supplementary Figure S4

- Supplementary Figure S5

- Supplementary Figure S6

- Supplementary Figure S7

**Figure legend**

**Figure S1** ZNF32 reduces autophagosome formation and inhibits autophagy initiation in SK-BR-3 and MDA-MB-231 cell lines. (A) Images of acridine orange (AO) staining and (B) RFP-LC3 transfection of SK-BR-3 and MDA-MB-231 cells with ZNF32 knock down or overexpression as detected using fluorescence microscopy. (C) Effect of ZNF32 knockdown or overexpression on LC3 II expression in SK-BR-3 and MDA-MB-231 cell lines.

**Figure S2** AKT/mTOR pathway is involved in the ZNF32-autophagy-cell death axis. (A) Flow cytometry revealed the effect of ZNF32 on MCF-7 cell death after a 24 h treatment with H_2_O_2_ (700 µmol/L) or diamide (500 µmol/L), 1 ng/ml EGF was used to enhance mTOR activation using a 3 h pretreatment.

**Figure S3** Expression of ZNF32 and LC-3 II in low- or high-grade pathological (A) luminal A and (B) luminal B breast cancer samples.


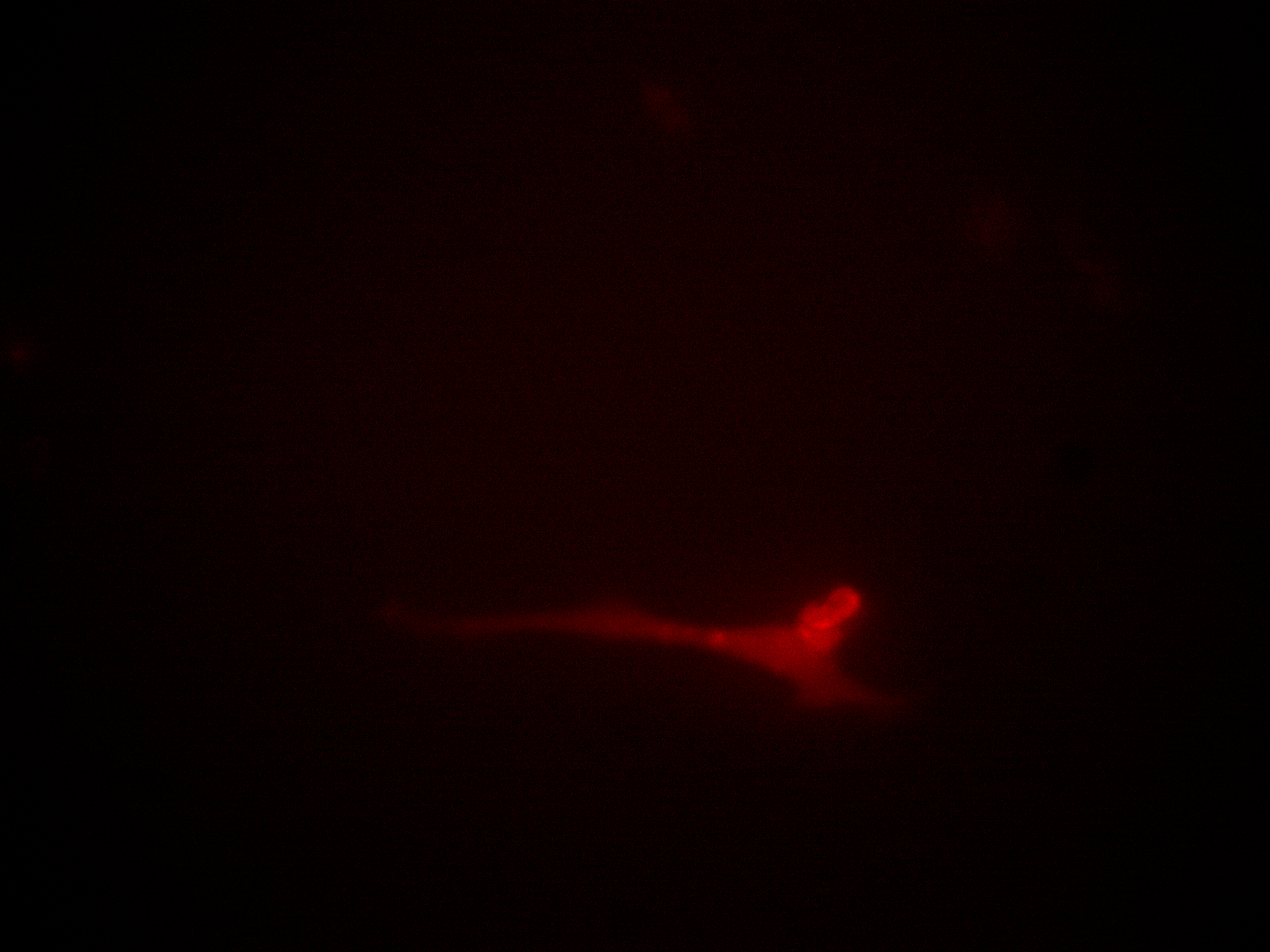

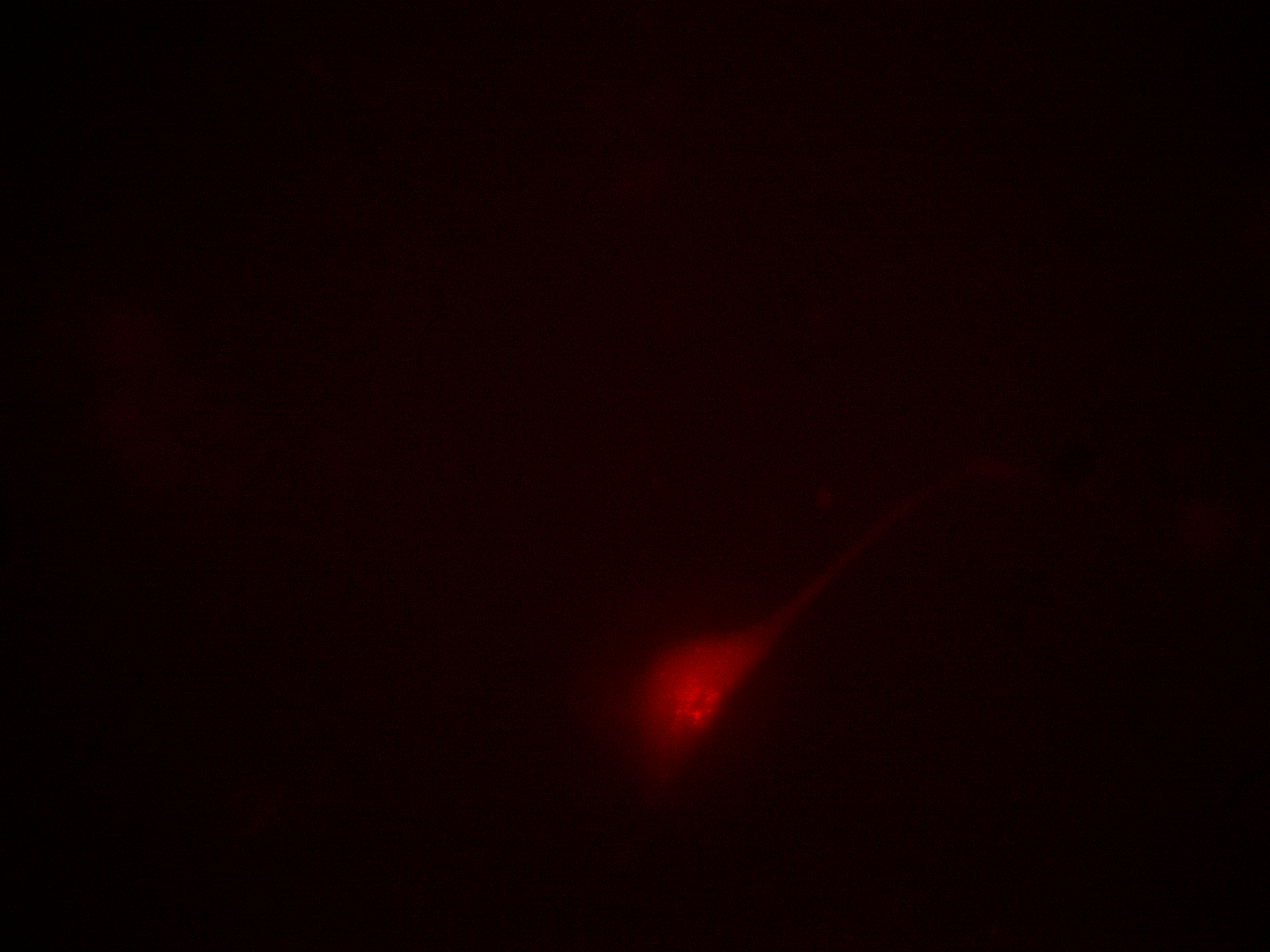

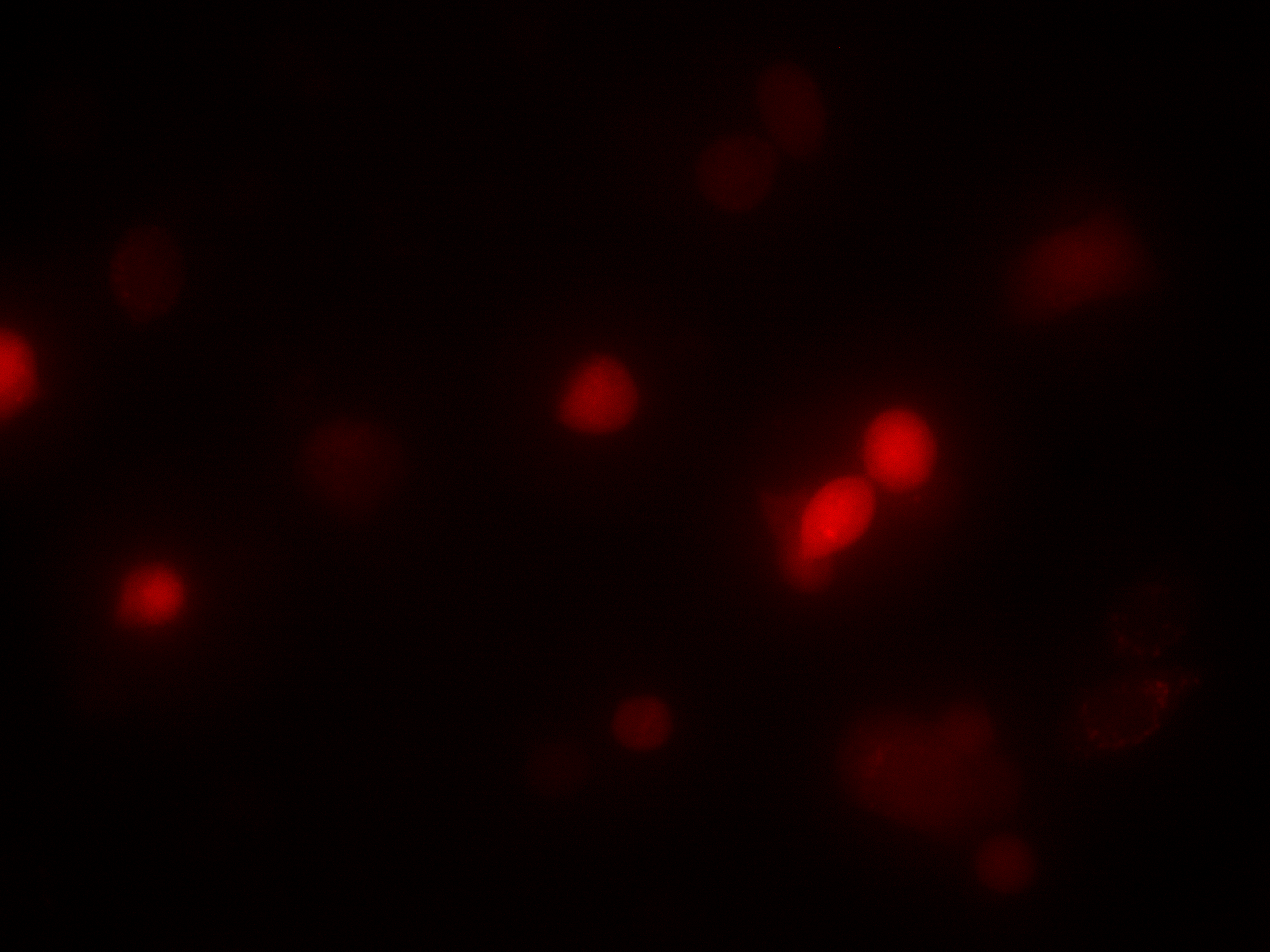

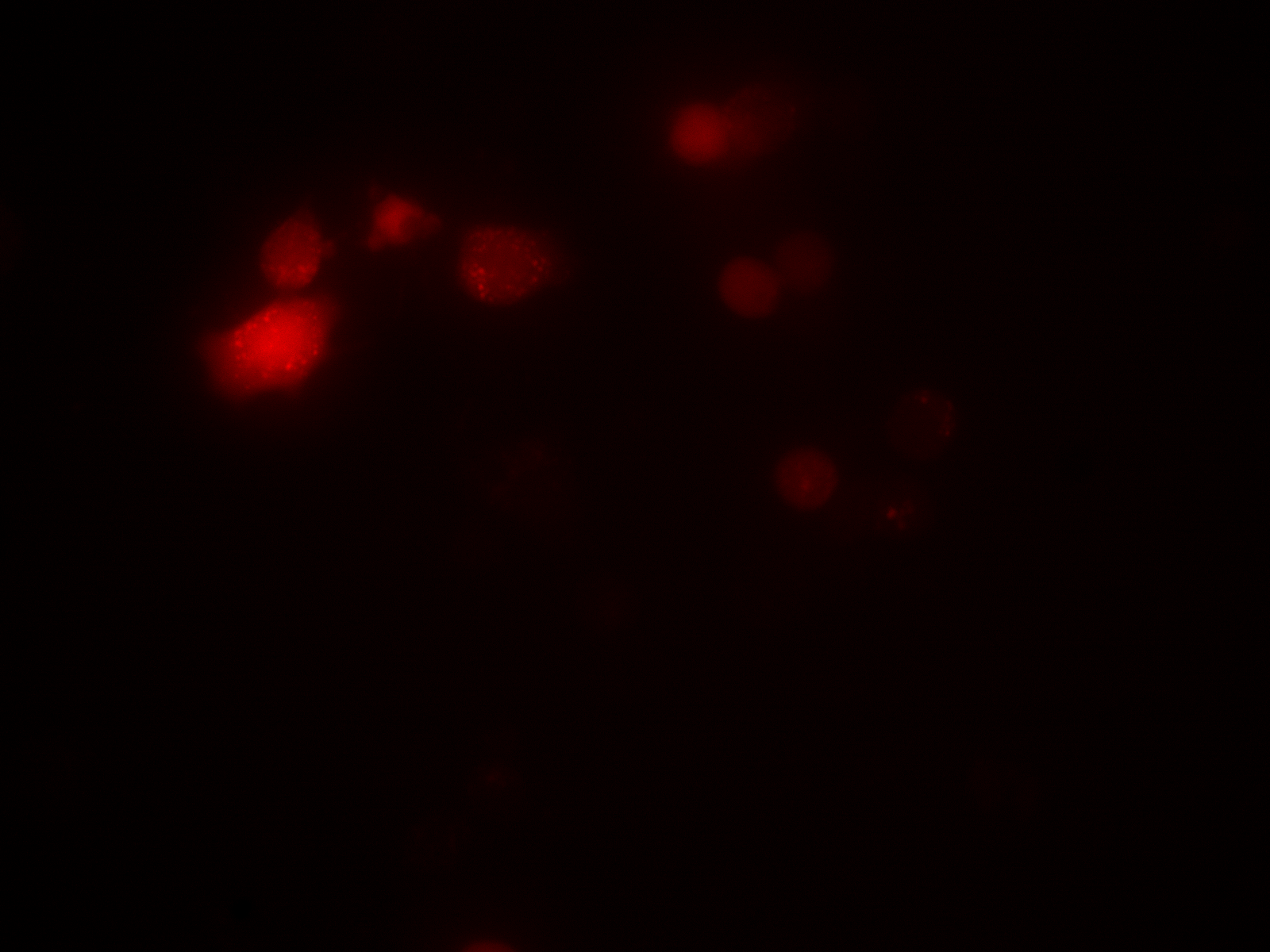

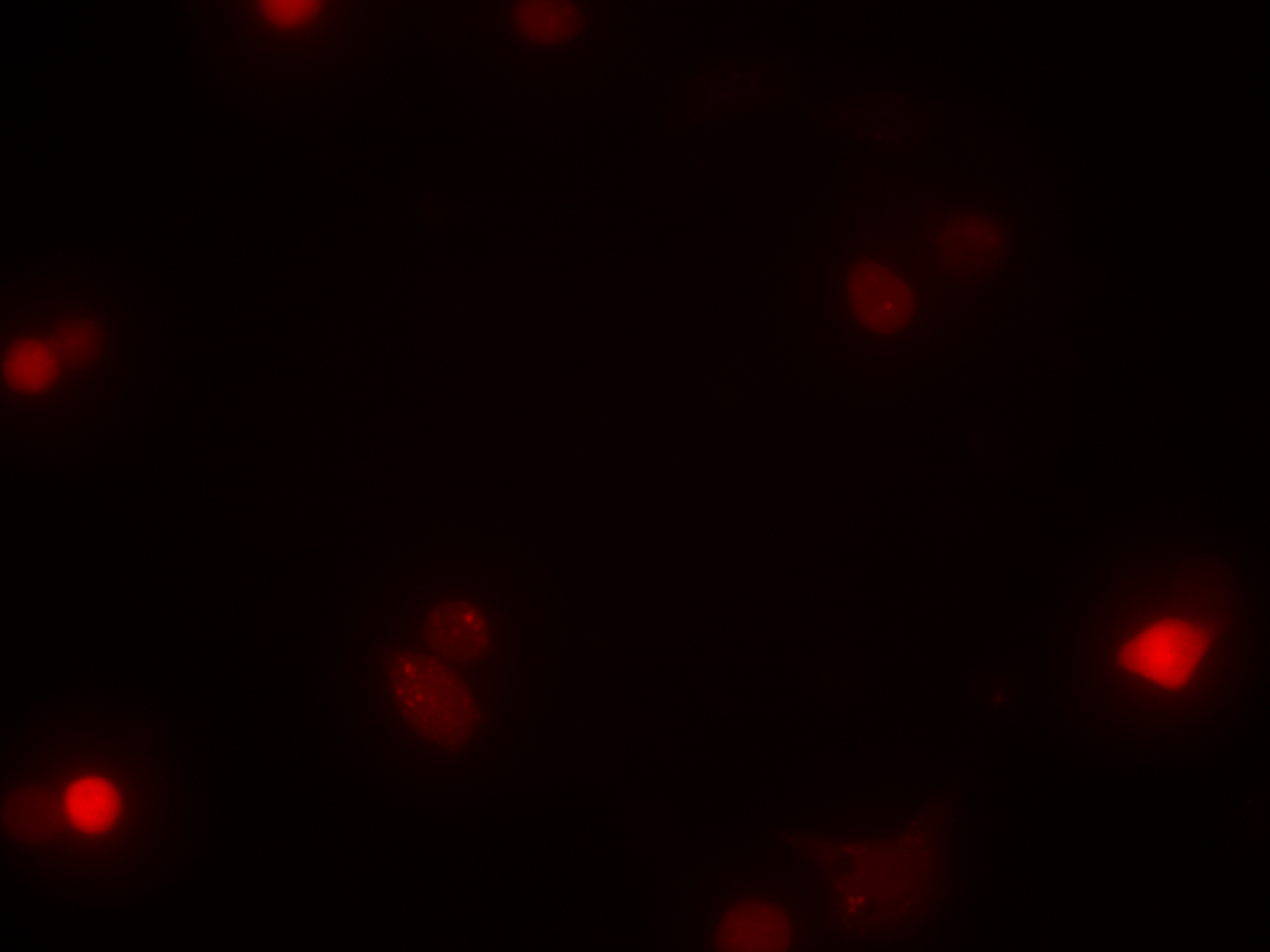

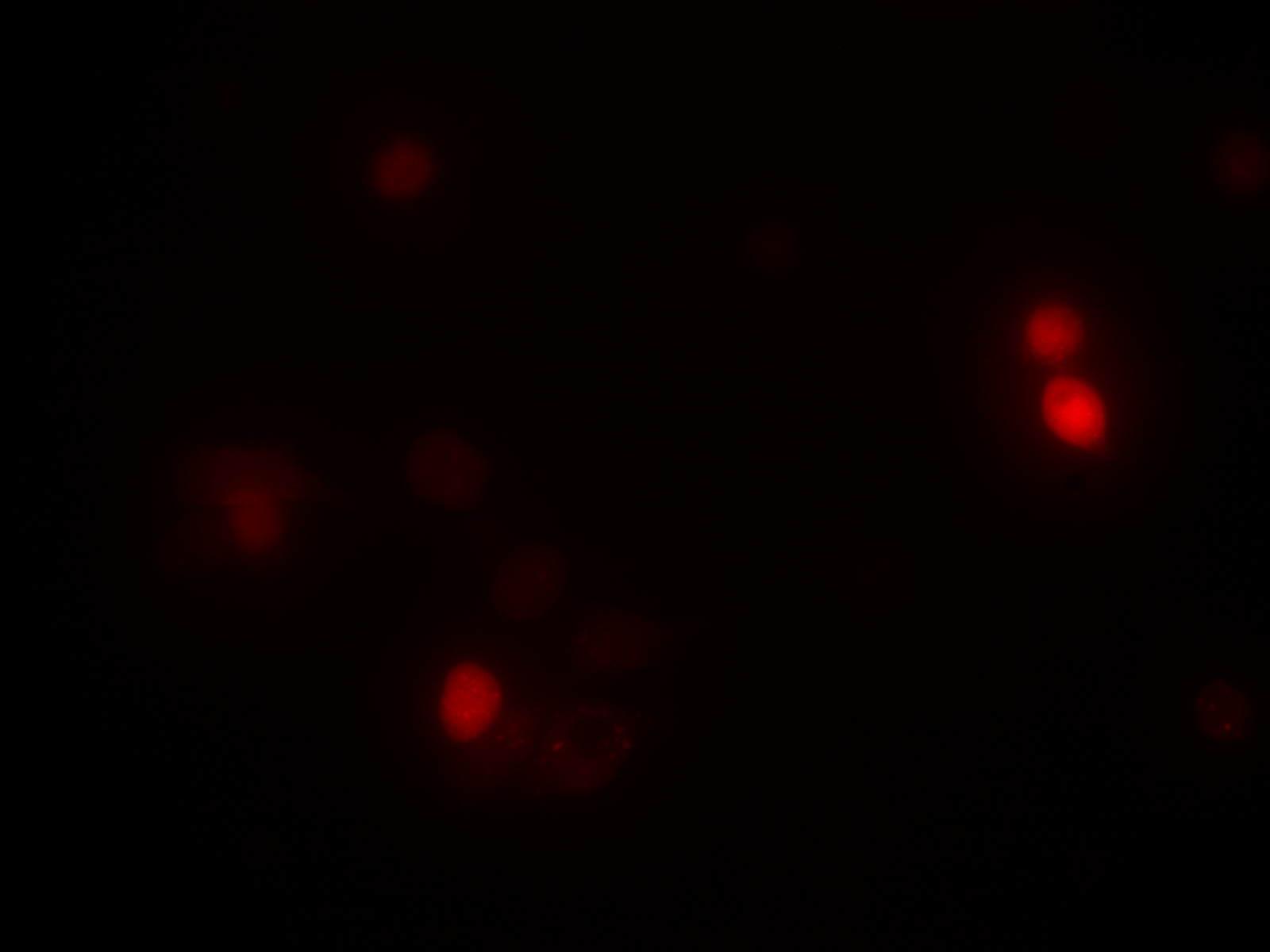

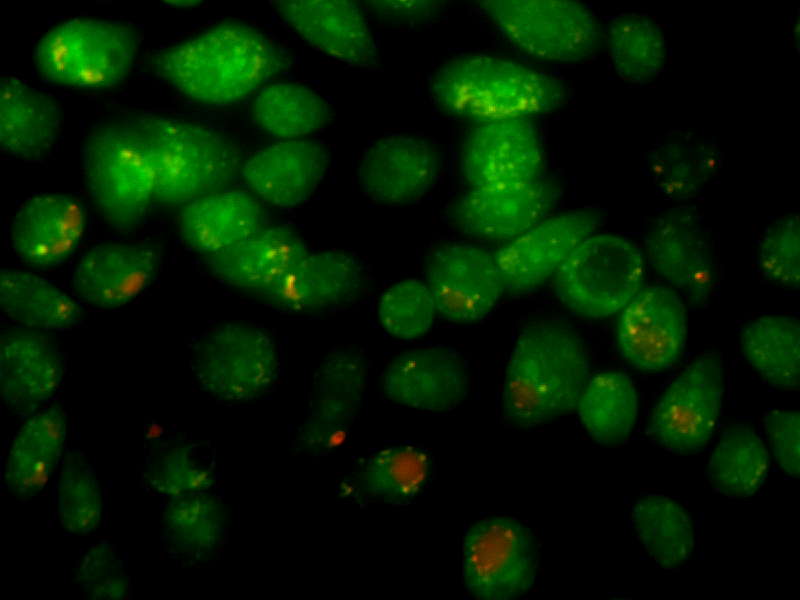

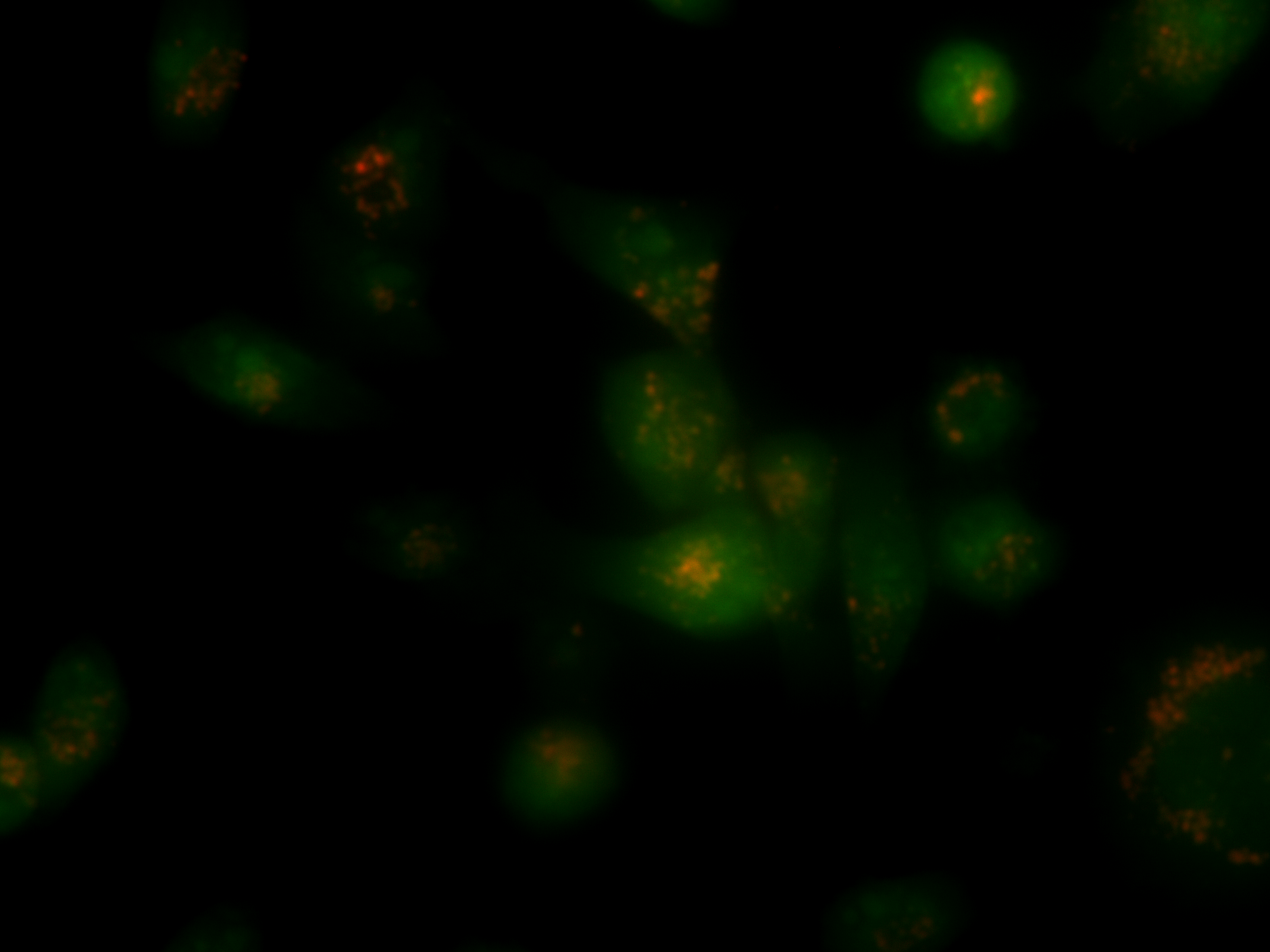

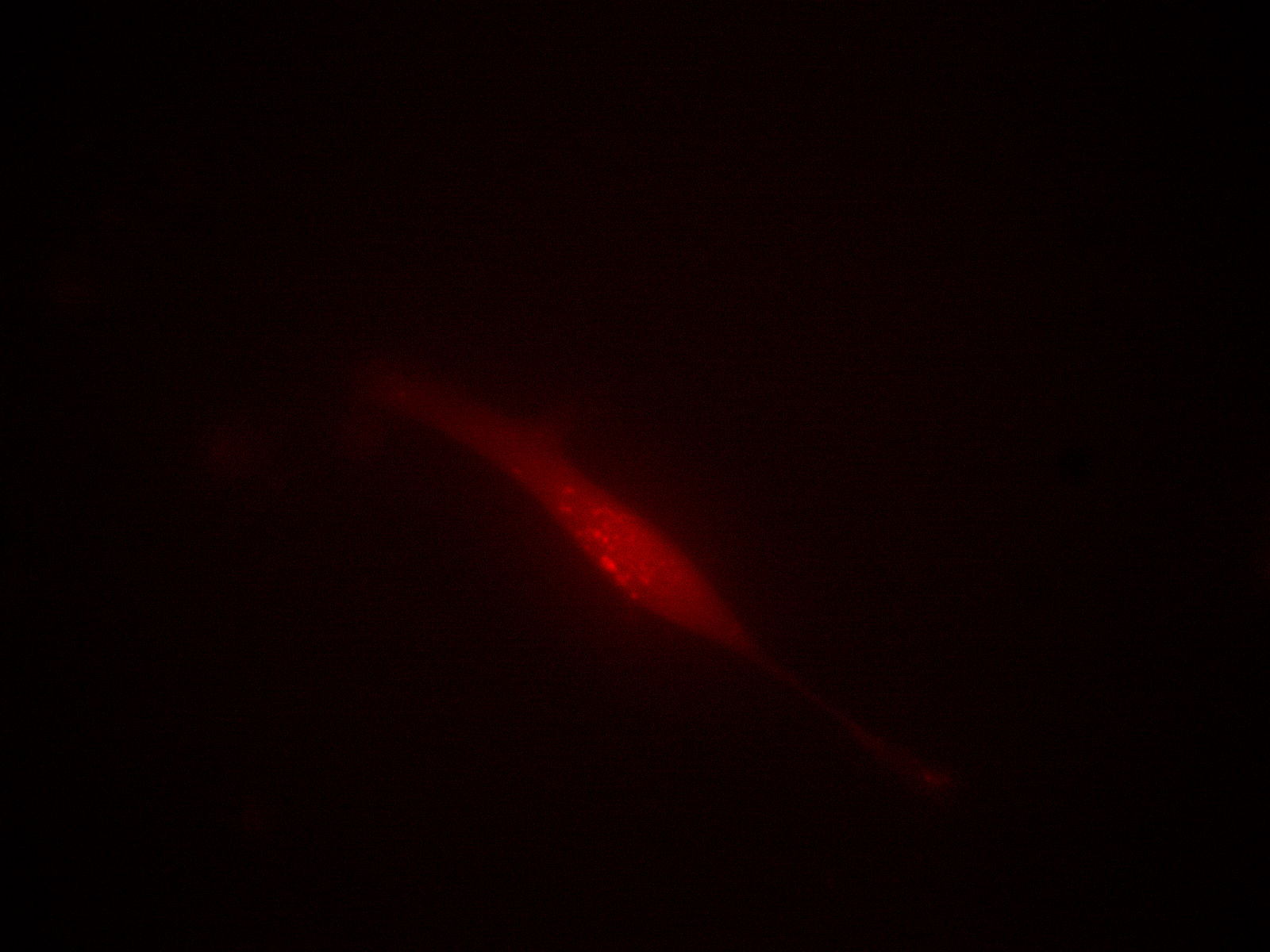

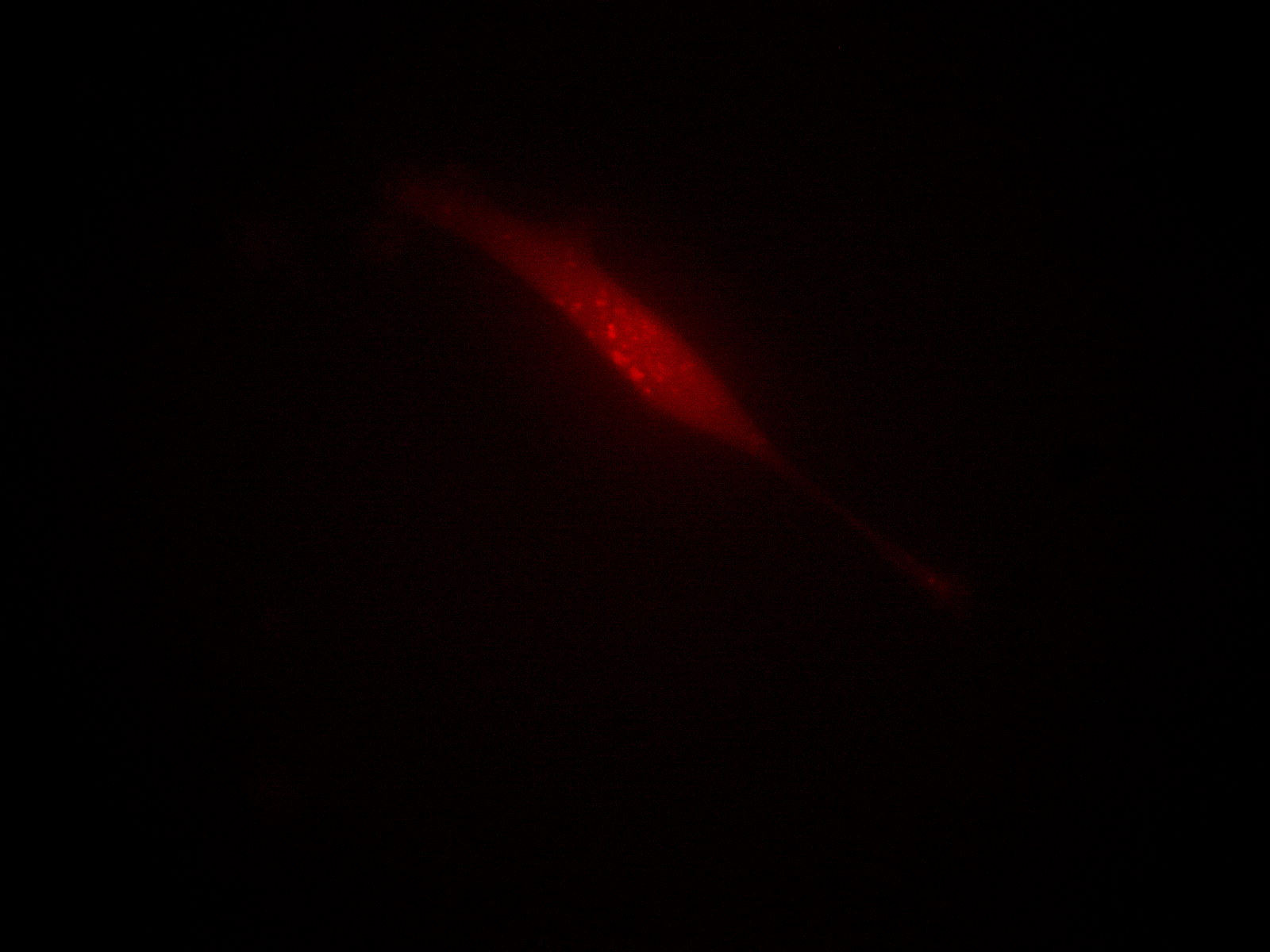

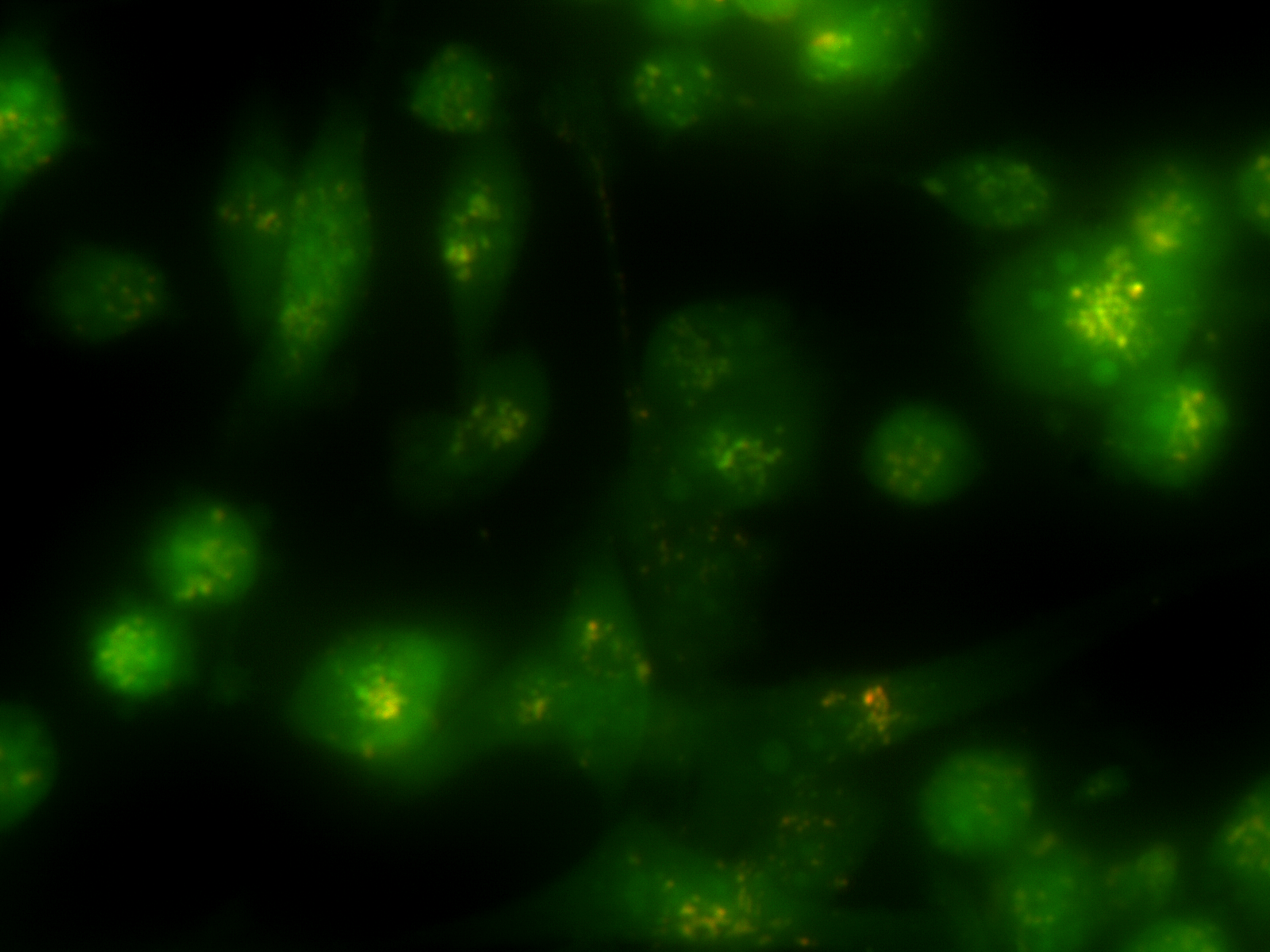

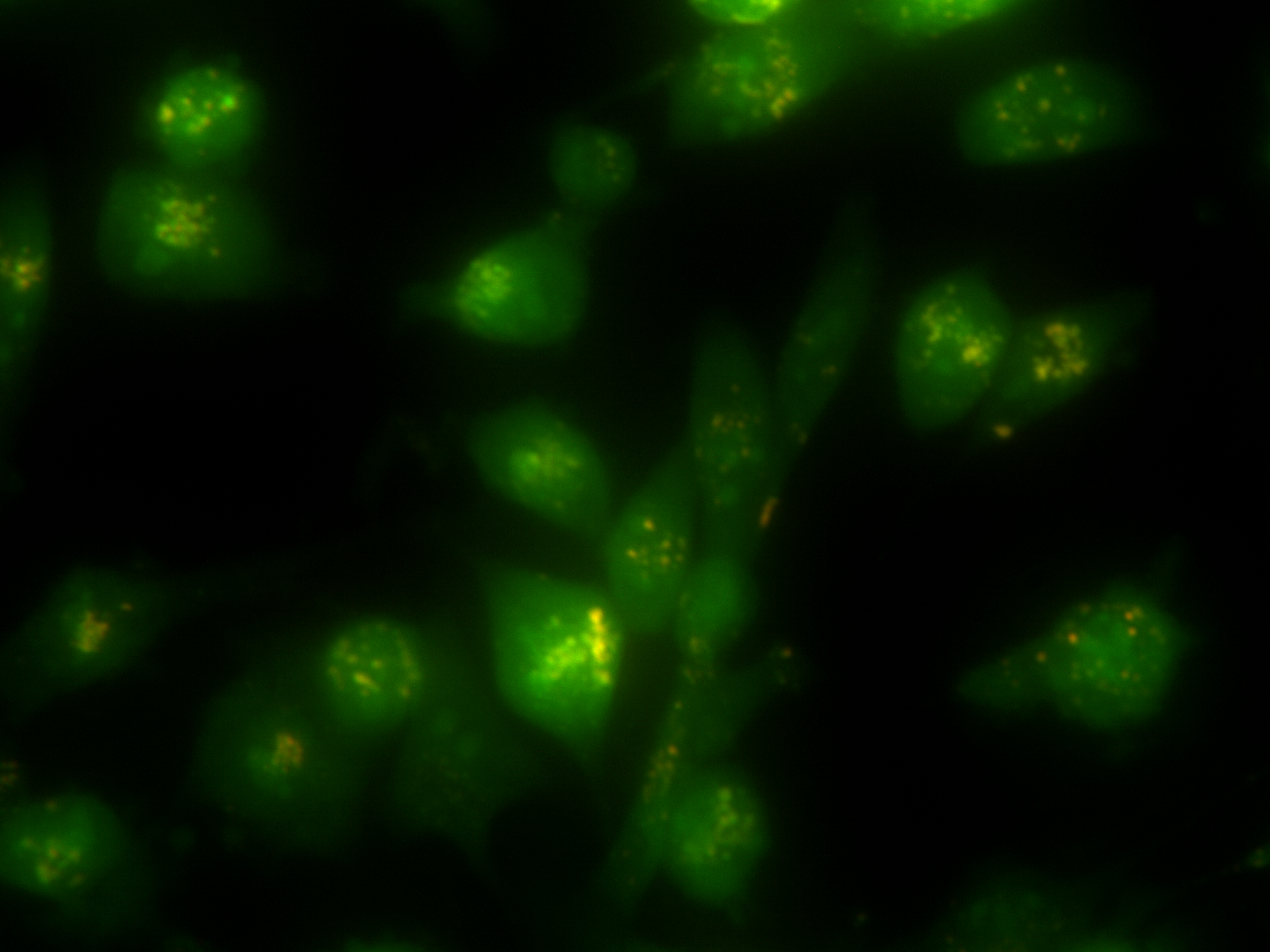

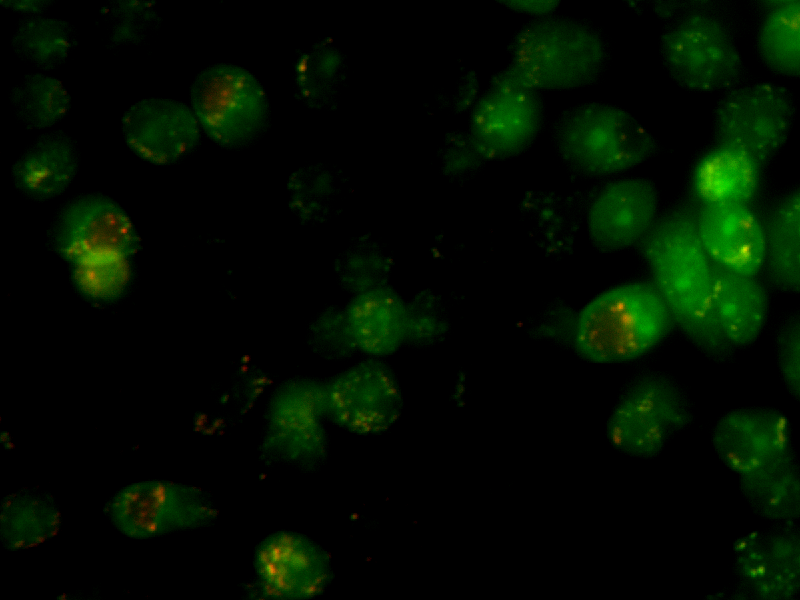

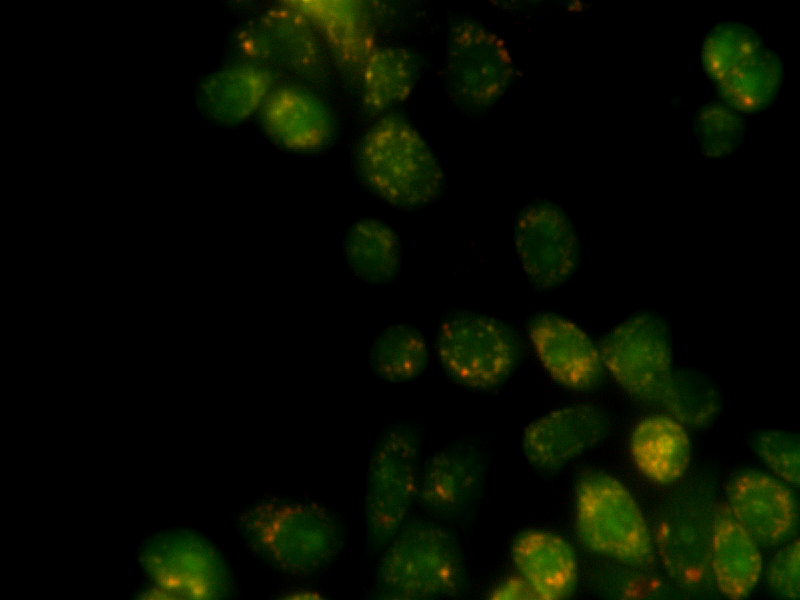

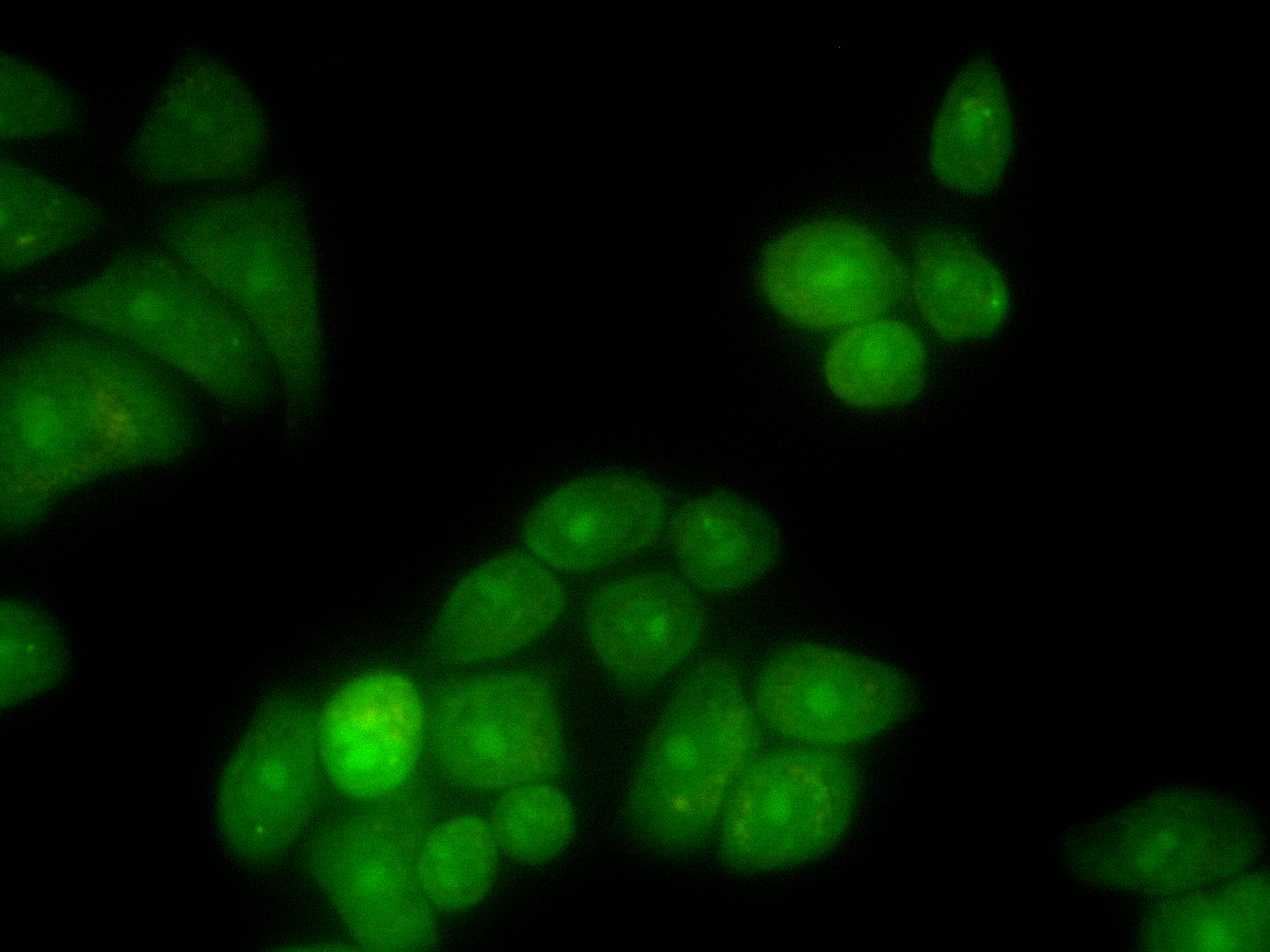


SK-BR-3

MDA-MB-231

Si-NC

ZNF32-KD

OE-vector

ZNF32-OE

Si-NC

ZNF32-KD

OE-vector

ZNF32-OE


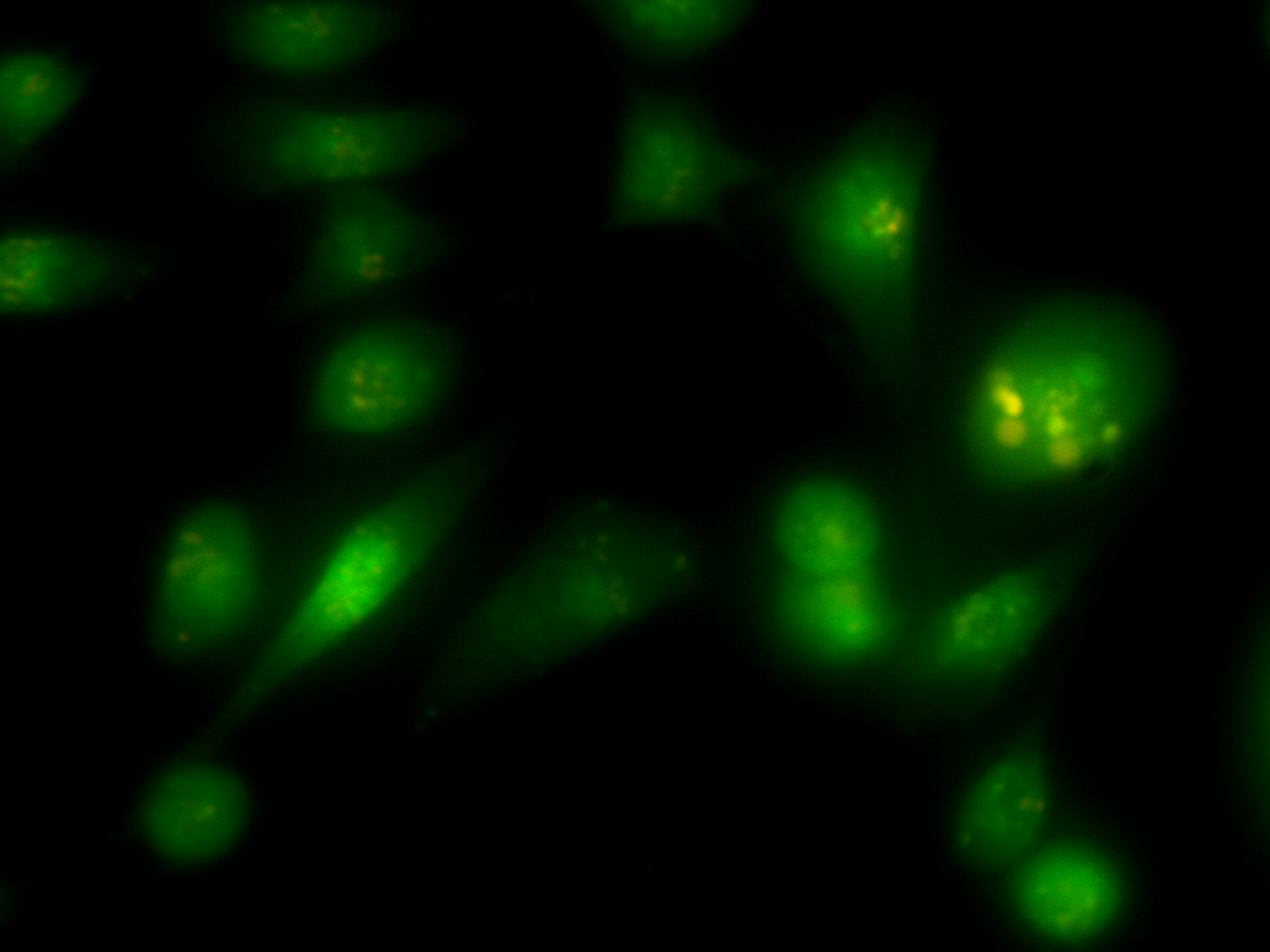


SK-BR-3

MDA-MB-231

LC3 I

II

SK-BR-3

MDA-MB-231

1:Si-NC

2:ZNF32-KD

3:OE-vector

4:ZNF32-OE

ZNF32


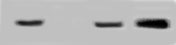

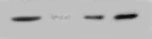

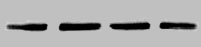

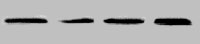

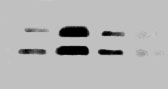

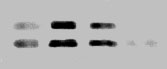


**C**

β-actin

1

1

2

2

3

3

4

4

1: Si-NC

2: ZNF32-KD

3:OE-vector

4:ZNF32-OE

**Figure S1 ZNF32 reduces autophagosome formation and inhibits autophagy initiation in SK-BR-3 and MDA-MB-231 cell lines.** (A) Images of acridine orange (AO) staining and (B) RFP-LC3 transfection of SK-BR-3 and MDA-MB-231 cells with ZNF32 knock down or overexpression as detected using fluorescence microscopy. (C) Effect of ZNF32 knockdown or overexpression on LC3 II expression in SK-BR-3 and MDA-MB-231 cell lines.

1: Si-NC

2: ZNF32-KD

3:OE-vector

4:ZNF32-OE

PI-A-subset 9.45%

A

PI-A-subset 8.77%

PI-A-subset 10.4%

count

PI-A-subset 12.2%

PI-A-subset 20.7%

PI-A-subset 11.7%

EGF

H2O2

control

PI-A-subset 15.2%

PI-A-subset 26.5%

PI-A-subset 15.8%

control

EGF

H2O2

PI-A-subset 21.4%

PI-A-subset 8.13%

PI-A-subset 27.5%

EGF

diamide

Si-NC

Si-NC

EGF

H2O2

diamide

ZNF32-KD

Si-NC

*

*

40

**B**

ZNF32-KD

EGF

H2O2

diamide

EGF

diamide

PI-A

ZNF32-KD

*

*

30

relative cell death (%)

20

10

control

EGF

H2O2

EGF + H2O2

diamide

EGF + diamide

0

**Figure S2** AKT/mTOR pathway is involved in the ZNF32-autophagy-cell death axis. (A) Flow cytometry revealed the effect of ZNF32 on MCF-7 cell death after a 24 h treatment with H_2_O_2_ (700 µmol/L) or diamide (500 µmol/L), 1 ng/ml EGF was used to enhance mTOR activation using a 3 h pretreatment.

7

LC3 II

**A**

6

**B**

ZNF32

LC3 II

ZNF32

6

5

ZNF32/LC3 II mean value

ZNF32/LC3 II mean value

4

5

4

3

3

2

2

1

1

0

0

-1

-1

low grade

high grade

low grade

high grade

**Figure S3 Expression of ZNF32 and LC-3 II in low- or high-grade pathological** (A) luminal A and (B) luminal B breast cancer samples.

**C**

**Figure S4**


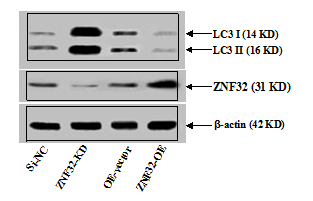


**E**


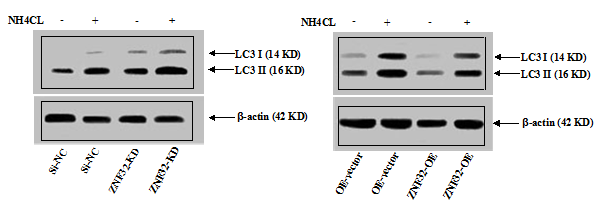


**Figure S4 ZNF32 reduces autophagosome formation and inhibits autophagy initiation in MCF-7 cells.** These are full length images of the cropped blot presented in the Figure 1C and 1E.


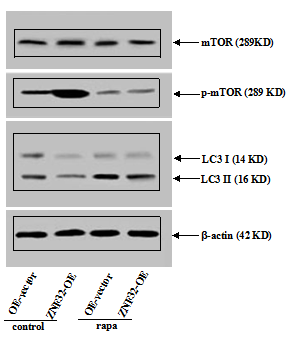

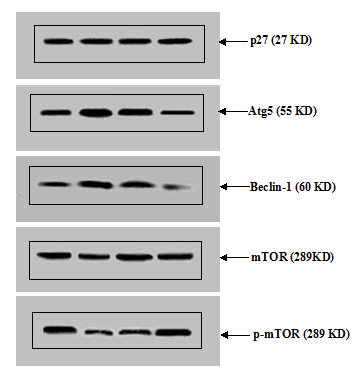


**A**

**Figure S5**

**B**


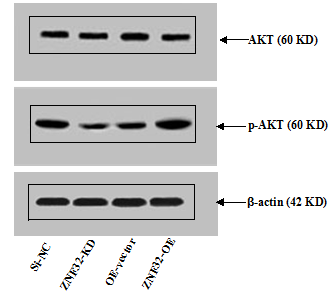


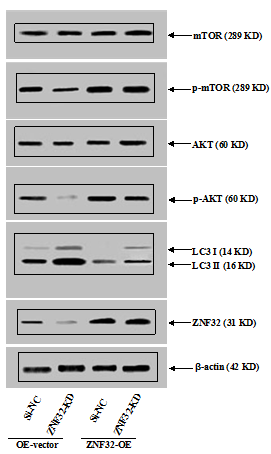


**E**


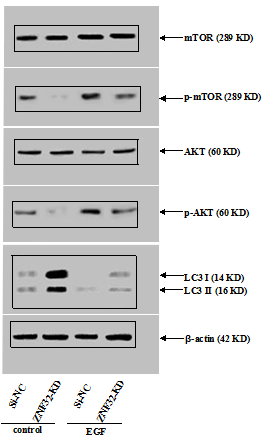


**C**

**Figure S5 ZNF32 inhibits autophagy initiation by activating the AKT/mTOR pathway.** These are full length images of the cropped blot presented in the Figure 2A/ 2B/2C and 2E.

**Figure S6**

**B**


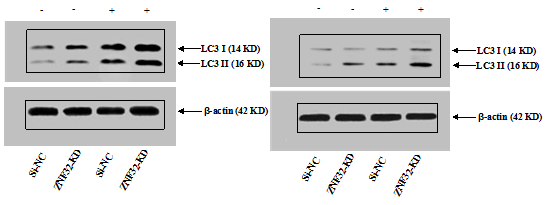


**F**


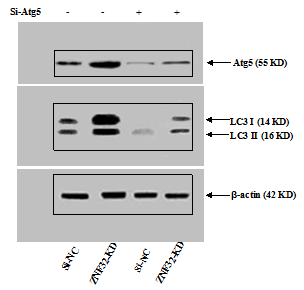


**Figure S6. ZNF32-associated autophagy participates in H2O2- and diamide-induced cell death.** These are full length images of the cropped blot presented in the Figure 3B and 3F.

**Figure S7**

**D**


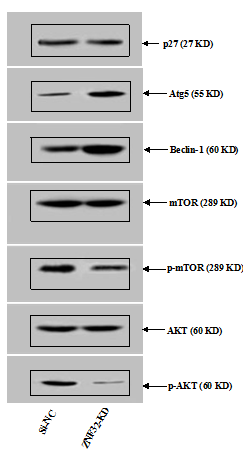


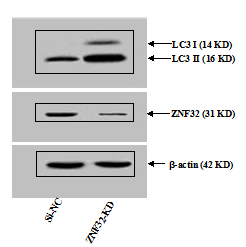


**Figure S7 ZNF32 is correlated with autophagy in both xenograft tumor-loaded mice and in breast cancer patients.** These are full length images of the cropped blot presented in the Figure 5D.
